# Advanced High-Strength Medium-Manganese Steels as an Alternative to Conventional Forging Steels: A Review

**DOI:** 10.3390/ma19010109

**Published:** 2025-12-28

**Authors:** Aleksandra Kozłowska, Anna Wojtacha

**Affiliations:** Department of Engineering Materials and Biomaterials, Faculty of Mechanical Engineering, Silesian University of Technology, 18A Konarskiego Street, 44-100 Gliwice, Poland; anna.wojtacha@polsl.pl

**Keywords:** advanced high-strength steel, automotive forgings, medium-Mn steel, retained austenite, impact toughness, crack resistance, fatigue behavior

## Abstract

This review highlights conventional forging steels and advanced medium-Mn steels containing retained austenite (RA), emphasizing their potential for industrial forging applications. Modern steels intended for forgings are required to combine strength, ductility, toughness and fatigue resistance with good hardenability and machinability at minimal cost. Medium-Mn multiphase steels fulfill these requirements by the strain-induced martensitic transformation (SIMT) of fine, lath-type RA, which can create a strength–ductility balance. Ferritic–austenitic steels provide high ductility with moderate strength, martensitic–austenitic steels show very high strength at the expense of ductility, and bainitic–austenitic steels achieve intermediate properties. Impact toughness and fatigue resistance are strongly influenced by the morphology of RA. The lath-type RA enhances energy absorption and delays crack initiation, while blocky RA may promote intergranular fracture. Low carbon (0.2–0.3 wt.%) combined with elevated manganese (3–7 wt.%) contents provides superior hardenability and machinability, enabling cost-effective air-hardening of components with various cross-sections. Advanced medium-Mn steels provide a superior mechanical performance and economically attractive solution for modern forgings, exceeding the limitations of conventional steel grades.

## 1. Introduction

The production of modern structural steels for forging applications requires continuous efforts to improve mechanical properties without increasing production costs [1,2,3]. Modern steels intended for forgings must combine high strength and impact toughness, while also meeting requirements for high fatigue strength, good machinability and low alloying costs. Furthermore, high hardenability is crucial as it ensures a uniform microstructure and stable mechanical properties through the entire cross-section of a component [4,5]. This is particularly important for the automotive industry, which produces components exposed to high loads such as crankshafts, suspension parts and chassis elements [6]. In modern vehicles, forgings are primarily used for chassis components, which are among the heaviest structural components and therefore provide the greatest potential for overall vehicle weight reduction. In the case of forgings, replacing traditional structural steels with new-generation materials such as Advanced High-Strength Steels (AHSS) may reduce the cross-section of components while maintaining the same load-bearing capacity. Another method is to optimize the chemical composition of steels intended for forgings by increasing the amount of low-density aluminum at the expense of other alloying elements. This leads to reduced fuel consumption and significantly lower harmful gasses emissions [7]. The introduction of new steel grades into production should contribute to a reduction in environmental impact. Reducing and shortening of heat treatment cycles help to save energy and decrease CO_2_ emissions. Additional heat treatment after forging, which involves reheating the element from room temperature, is expensive and time-consuming. Modern steels should exhibit good hardenability, which allow air-cooling, instead of quenched in water or oil. Such an approach simultaneously reduces the consumption of cooling media and energy. Shorter and more efficient steelmaking and forging processes are essential for the reduction of environmental impact and will also contribute to lower production costs [8,9]. Meeting these diverse requirements simultaneously is a major challenge and often demands alternative material and technological solutions compared to those ones currently in use.

In the case of currently used conventional steels for forgings, the most common are steels with tempered martensite microstructure and precipitation-hardened pearlitic–ferritic steels. These steels meet only selected requirements crucial for forgings. Ferritic–pearlitic steels are characterized by high fatigue strength and good machinability, while exhibiting relatively low impact toughness. Martensitic steels show high strength properties, particularly impact toughness compared to the ferritic–pearlitic structure. The main issue of conventional martensitic steel is the high content of alloying elements, such as expensive chromium and an elevated carbon content required to achieve adequate hardenability and strength properties. Bainitic steels are used less frequently than ferritic–pearlitic steels mainly due to challenges in ensuring adequate microstructural homogeneity [10,11]. One of the most rapidly developing groups of steels for forgings includes ferritic–bainitic and ferritic–martensitic steels, which offer a favorable combination of strength and ductility. However, the thermomechanical processing routes of such steels involve several stages.

One of the most promising alloys for forging applications are AHSS with a multiphase microstructure containing retained austenite (RA). Such steels may offer a beneficial combination of high strength, impact toughness and fatigue strength under various loading conditions [12,13,14,15]. Among the AHSS group, medium-Mn steels containing 3–12 wt.% Mn show the greatest potential for application in automotive forgings [16,17,18,19,20]. So far, multiphase steels containing RA have been successfully used for the production of automotive sheets. However, this type of steel has not been applied in the industry for the production of forgings. Research on multiphase steels containing RA intended for forgings has been conducted for several years by Japanese research groups [2,4,21,22]; however, it has focused on steels with a manganese content not exceeding 1.5 wt.%. One of the first studies on the effect of the forging process on the mechanical properties of steel with the chemical composition 0.42C-1.51Mn-1.47Si-0.5Cr-0.48Al-0.2Mo-0.05Nb was carried out by Sugimoto et al. [23]. The resulting microstructure consisted of a small amount of polygonal ferrite, bainitic ferrite and RA in the form of blocky grains and thin films located between the bainitic ferrite laths. The tested steel showed a favorable combination of mechanical properties: YS = 880 MPa, UTS = 1390 MPa and KV = 88 J. The main problem of the described solution is the difficult machinability of steel due to an increased carbon content and the heterogeneity of the microstructure related to the presence of polygonal ferrite and blocky grains of RA.

Forged components remain crucial in many industrial sectors requiring a favorable balance of strength, ductility and cost effectiveness. Conventional steels used for forgings meet only limited criteria. Future-oriented medium-Mn steels containing RA with complex multiphase microstructures offer promising alternatives for conventional steels, providing enhanced mechanical properties and improved processability. The advantageous properties that offer multiphase medium-Mn steels confirm the need for a comprehensive review of the current state of knowledge and the prospective development directions for steels intended for forging applications. This review not only systematizes knowledge on conventional steels used for forgings, but also compares it with the current state of research on medium-Mn steels containing RA and highlights their potential for implementation in the forging industry. The particular value is the identification of key trade-offs between strength, ductility, impact toughness and fatigue strength, which are crucial for the design of forging steels. The review also emphasizes the role of microstructure control, including RA stability, as a factor enabling tailoring of performance properties. From an industrial point of view, the review indicates that medium-Mn steels containing RA can represent a promising alternative to conventional forging steels.

## 2. Conventional Steels for Forging Applications

The conventional steel grades used for the production of forged components are summarized in Table 1. Conventional processing routes of forgings involve several heat treatment operations or multi-step cooling. Despite their widespread industrial use, conventional forging practices often face limitations in achieving uniform properties in large or complex components.

### 2.1. Steels with a Tempered Martensite Microstructure

**Figure 1:**
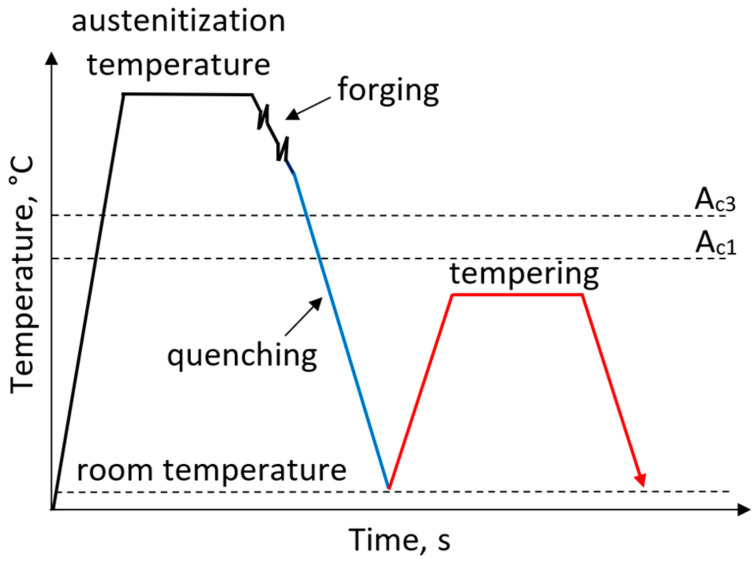
Processing schedule for forgings with a microstructure of tempered martensite.

Currently steels with a tempered martensitic microstructure are widely used for forgings. Forged components manufactured through hot forging and subsequent heat treatment exhibit high mechanical properties and high impact toughness. The heat treatment routes for such steels includes austenitization, hot forging followed by quenching to room temperature and subsequent high-temperature tempering performed in a temperature range of 500–650 °C (Figure 1). However, single-stage tempering is often insufficient for large forged components. The application of multi-step tempering enables the achievement of the desired mechanical properties and microstructure homogeneity [25,26,27,28,29].

High-temperature tempering is applied to reduce residual stresses, thereby decreasing brittleness and improving both ductility and impact toughness. Tempering of martensitic steels is a complex process associated with carbide precipitation and recovery of dislocation structures within the supersaturated martensitic laths [30]. These processes result in a marked decrease in hardness and strength, accompanied by a considerable improvement in ductility and impact toughness [31,32]. However, the achievement of high strength is often accompanied by reduced fatigue resistance and difficult machinability. The main disadvantages of steels with a tempered martensite microstructure include their high alloying content and the complexity of the manufacturing processes, which significantly increases production costs [25,26]. To ensure high hardenability, strength and impact toughness, increased amounts of alloying elements such as Cr, Ni and Mo as well as C are introduced into the steel. Selected chemical compositions and corresponding mechanical properties of commercial martensitic steels are presented in Table 2 [27].

### 2.2. Steels with a Dislocated Martensite Microstructure

**Figure 2:**
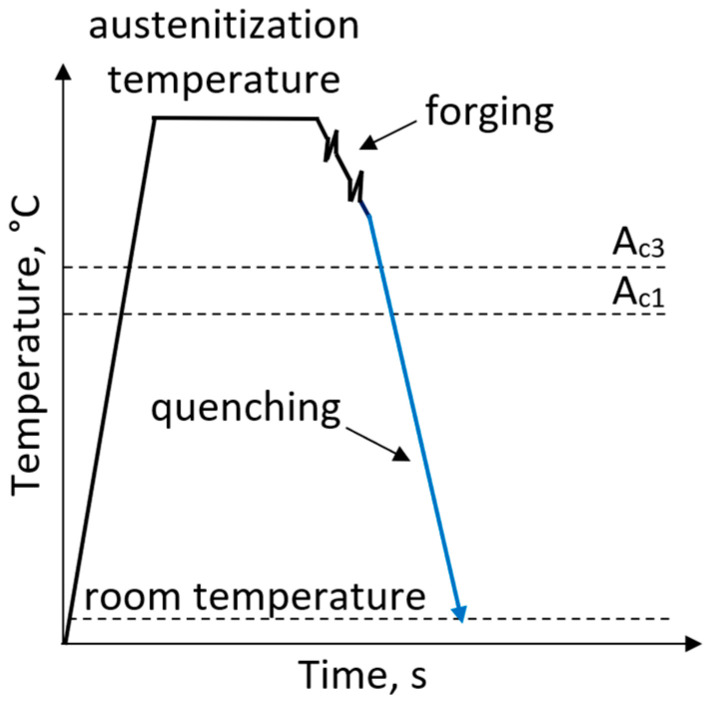
Processing schedule for forgings with a microstructure of dislocated martensite.

Steels with a dislocated martensite microstructure allow for the manufacturing of high-strength, low-alloyed forgings that exhibit good ductility. The processing route of these steels includes water quenching immediately after forging, without subsequent tempering (Figure 2). The mechanical properties of steels with a dislocated martensite microstructure are strongly influenced by the austenite-to-martensite transformation which should proceed without generating excessive internal stresses. The transformation of the face-centered cubic (FCC) structure characteristic for austenite into the body-centered cubic (BCC) structure characteristic for martensite via spontaneous shear requires a substantial strain, which is accommodated by introducing a high density of defects [33]. Steels with a high carbon content (>0.6 wt.%) or a low martensitic transformation temperature typically exhibit internal twins as the dominant defects. The twinned martensite shows very high strength but is very brittle. Therefore, the carbon content in steel with dislocated martensite microstructure should be below 0.6 wt.% to develop dislocations as the primary defects [34]. The chemical composition of martensitic steels must reflect a compromise between achieving high hardenability and enabling the formation of self-tempered dislocation martensite. Therefore, this limits their use to products with a cross-section not exceeding 30 mm [35].

### 2.3. Steels with a Ferritic–Pearlitic Microstructure

**Figure 3:**
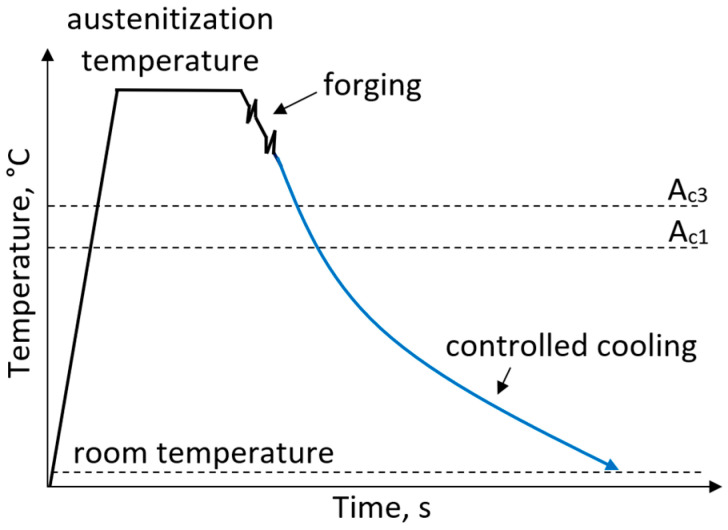
Processing schedule for forgings with a ferritic–pearlitic microstructure.

The first studies on ferritic–pearlitic microalloyed steels containing V and Nb microadditions were conducted between 1970 and 1980 and demonstrated that high mechanical properties could be achieved for forgings by allowing direct cooling from the final forging temperature (Figure 3). The chemical composition of ferritic–pearlitic steels is composed of <0.5 wt.% C, <2 wt.% Mn and <0.9 wt.% Si together with microalloying additions of Ti, V and Nb in total amounts below 0.1 wt.% [28]. Ti and V microadditions form TiC, TiN, VN and V(C,N) precipitates, which inhibit austenite grain growth during austenitization. During heating and austenitization, Ti and V-containing compounds should not fully dissolve in the solid solution, because this promotes undesirable grain growth. This mechanism promotes grain refinement improving both the yield strength and the impact toughness of the steel [36]. For forgings with complex shapes, the proper design of process parameters is challenging. To ensure the desired kinetics of phase transformations, it is necessary to maintain appropriate hot deformation parameters. Moreover, cooling rates must be adjusted to the varying cross-sections of the forgings to achieve microstructure homogeneity [37,38,39,40]. Rasouli et al. [41] investigated the effect of the post-forging cooling rate on the microstructure and mechanical properties of the microalloyed 30MSV6 steel grade. They observed that the cooling rate after forging influences the size and distribution of ferrite and pearlite as well as the thickness and arrangement of pearlite lamellae. With increasing cooling rate, the resulting ferrite–pearlite microstructures were observed to transform into acicular ferrite, bainite or martensite. Selected chemical compositions and corresponding mechanical properties of ferritic–pearlitic steels obtained using different cooling media are presented in Table 3.

Compared to steels with a microstructure of tempered martensite, ferritic–pearlitic microalloyed steels exhibit significantly lower impact toughness, which limits their application. However, they are characterized by higher fatigue strength and overall good machinability and do not require implementation of additional heat treatment [44,45].

### 2.4. Steels with a Bainitic Microstructure

**Figure 4:**
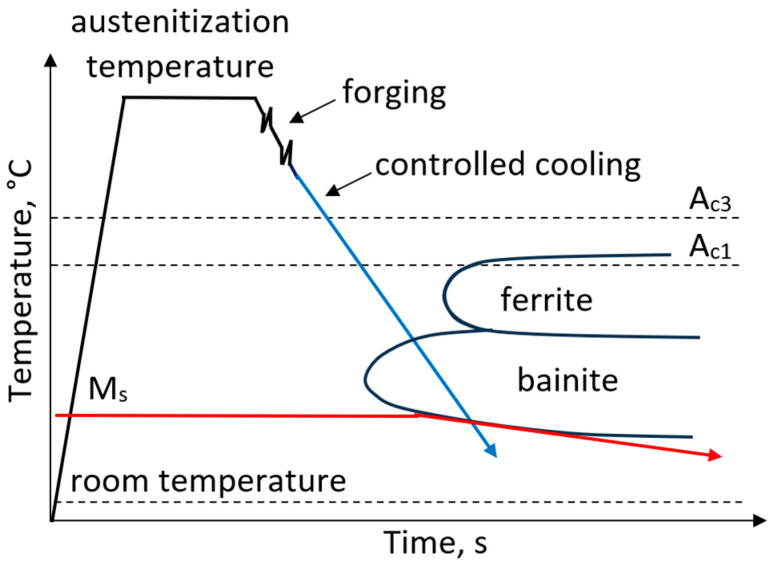
Processing schedule for forgings with a bainitic microstructure.

An intermediate range of mechanical properties between ferritic–pearlitic and martensitic steels can be achieved for bainitic steels. Bainitic steels for forgings have chemical compositions similar to microalloyed ferritic–pearlitic steels. However, to increase hardenability, Mo or Cr in a content of 0.2–1 wt.% and V with a content up to 0.15 wt.% are added to the steel [46,47,48]. The processing route of forged components with a bainitic microstructure includes austenitization, hot deformation and subsequent controlled cooling to obtain a bainitic microstructure (Figure 4). The hot deformation parameters strongly affect the recrystallization process and consequently, determine the final microstructure of forged component. Silveira et al. [49] observed that forging processes performed at lower temperatures increased the fraction of polygonal ferrite, resulting in a microstructure heterogeneity, and promoted martensitic transformation during cooling. On the other hand, forging processes conducted at higher temperatures favored the formation of undesirable granular bainite [50,51,52]. Tailoring the bainite morphology is crucial for achieving high mechanical properties of bainitic forgings [53,54,55,56,57].

The application of continuous cooling after forging enables a reduction in manufacturing costs as it eliminates the need for additional heat treatment operations. Bainitic steels are used significantly less frequently than ferritic–pearlitic steels, primarily due to challenges in ensuring adequate microstructural homogeneity [10]. Selected chemical compositions and corresponding mechanical properties of bainitic steel for forgings are presented in Table 4.

Lisiecka-Graca et al. [58] reported that nanobainitic steels exhibit a high potential for forging applications due to their advantageous combination of strength, ductility and in-use properties. However, for forgings with large cross-sections, the prolonged isothermal heat-treatment required to obtain a uniform nanobainitic microstructure makes this approach uneconomical. Nevertheless, nanobainitic steels can be successfully used for forgings with small cross-sections [59].

### 2.5. Steels with Ferritic–Bainitic and Ferritic–Martensitic Microstructure

**Figure 5:**
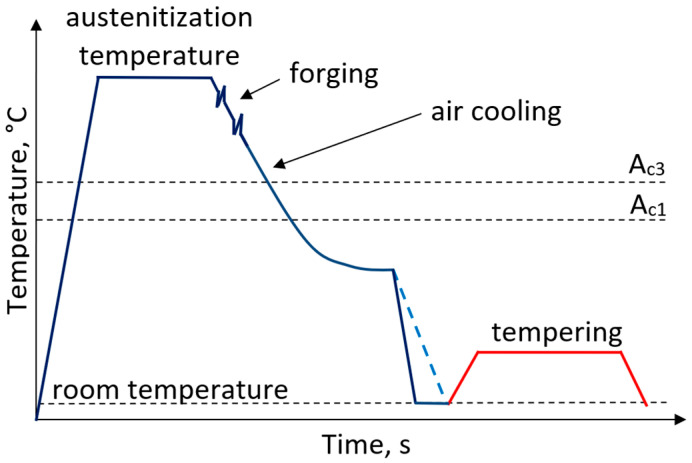
Processing schedule for forgings with a ferritic–bainitic microstructure (dashed line) and ferritic–martensitic microstructure (solid line).

Another group of forging steels includes dual-phase ferritic–bainitic and ferritic–martensitic steels, which undergo a two-step cooling process immediately after forging, followed by a low-temperature tempering treatment (Figure 5). These steels have chemical compositions similar to ferritic–pearlitic steels. The two-stage cooling process after forging results in a dual-phase microstructure consisting of soft, polygonal ferrite grains (formed during the initial cooling) and hard phases such as bainite and martensite islands (formed during the rapid second-stage cooling). The additional tempering carried out at about 400 °C applied for ferritic–martensitic steel leads to the tempering of the hard phases and additional dispersion strengthening of the ferritic matrix [60]. This multi-step heat treatment significantly improves strength and ductility, but at the same time, it significantly increases production costs.

Differences in the type and volume fractions of the individual microstructural constituents in ferritic–bainitic and ferritic–martensitic steels affect the resulting mechanical properties (Table 5). Saeidi et al. [61] investigated two dual-phase steels with a chemical composition of 0.40C-0.62Mn-0.29Si-0.73Cr-1.77Ni-0.21Mo, which were characterized by ferritic–martensitic and ferritic–bainitic microstructures containing 34 vol.% of ferrite and two reference steels with fully bainitic and fully martensitic microstructures. The results indicated that ferritic–bainitic steel exhibits lower strength than the bainitic steel due to the presence of softer ferrite. The study demonstrated that ferrite in the ferritic–martensitic steel exhibited greater strain hardening and higher strength than ferrite in the ferritic–bainitic steel. Authors also observed that the dual-phase ferritic–bainitic microstructure exhibited a higher impact toughness and total elongation compared to the fully martensitic or bainitic steels. They also noted that the presence of ductile ferrite delays microcrack propagation, thereby enhancing resistance to brittle fracture.

## 3. Advanced Steels for Forging Application

### 3.1. Influence of Retained Austenite on the Mechanical Behavior of Multiphase Steels

The application of advanced multiphase steels containing RA in the production of forgings addresses the current requirements of the global automotive industry [62,63]. Steels intended for forgings are expected to show high strength under static, dynamic and cyclic types of load [63,64]. A beneficial combination of mechanical properties can be achieved due to the presence of RA in the microstructure. The metastable RA enables a simultaneous increase in the strength and ductility of multiphase steels, and it is called TRIP effect [65,66,67]. Such effect has been extensively documented in the literature on multiphase steels intended for automotive sheets [65,66,67,68,69]. Some fraction of the metastable RA transforms into martensite during manufacturing operations such as drawing, bending or stamping, which allows for good formability while maintaining high strength properties [15,70]. The remaining fraction of RA may transform into martensite during service conditions or vehicle crash events. The strain-induced martensitic transformation (SIMT) of RA enhances the energy absorption capacity of the component contributing to increased crashworthiness and improved safety of vehicle users [71]. The SIMT is widely applied in flat products, where a uniform and controlled stress state and RA can be easily achieved. This mechanism in flat products has been extensively studied by numerous researchers. In the case of products with different shapes (e.g., forgings), heat treatment and deformation conditions are more complex. Large cross-sections and complex geometries make it difficult to precisely control the stability of RA throughout the cross-sectional profile, which is more easily achieved in flat products. The mechanical response of the steel containing RA is directly linked to its fraction and stability. There are several factors affecting the stability of RA, which can be divided as internal and external. The internal factors include chemical composition (typically a Mn and C content), morphology of RA (granular or lath-type), its grain/lath size and a type of matrix surrounding the RA [72,73]. The major external factors include stress state, deformation temperature and strain rate [74,75]. All these factors should be taken into account when designing the processing routes of multiphase steels containing RA.

**Figure 6:**
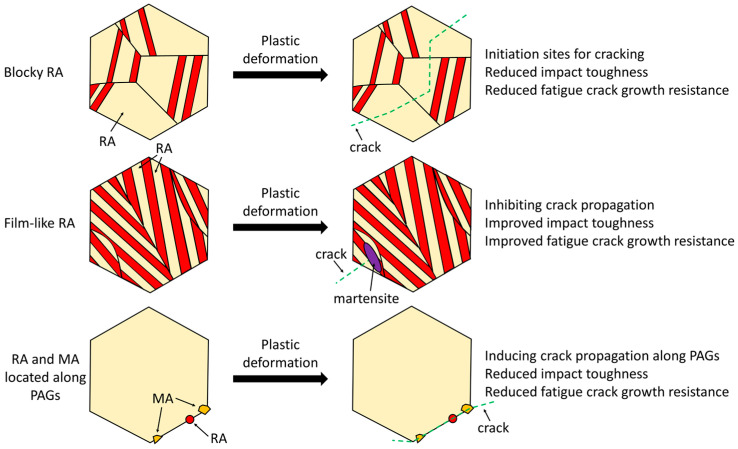
Schematic illustration of the fracture mechanisms in multiphase steels depending on the morphology of RA.

The role of RA in forgings differs from that observed in flat products. Forgings are not subjected to cold forming processes; thus the RA in the final product remains untransformed. Therefore, the benefits resulting from the present of RA in the microstructure of forgings are limited to the loads resulting from their service conditions. Numerous research studies reported the beneficial effect of RA on the mechanical response of multiphase steels under uniaxial tensile loading [15,68,75,76,77]. The gradual transformation of RA into martensite plays a significant role in the deformation process ensuring a high work hardening rate and contributing to delayed necking. However, the effect of RA on impact toughness and crack resistance, which are particularly important properties for forgings, remains inconclusive. It was reported that the RA enhances impact toughness by absorbing the energy resulting from the applied load [78]. On the one hand, RA prevents the formation of microcracks by providing local plasticity. The martensitic transformation of RA during plastic deformation contributes to the absorption of the energy for crack propagation and the decrease in the intensity of crack growth via the transformation-induced local strengthening and crack closure mechanism [79,80]. On the other hand, some studies have reported that RA may deteriorate impact toughness and fatigue crack resistance. Gulbay et al. [69] reported that the presence of martensite–austenite (MA) islands and RA showing low stability facilitates intergranular fracture through the development of a brittle network along prior austenite grains (PAGs) in carbide-free bainitic steel. They observed a cleavage fracture after the Charpy impact tests caused by enhanced debonding along PAGs facilitated by MA islands. Gao et al. [79] also reported that MA islands and blocky RA located at PAGs have a negative effect on the mechanical behavior of bainitic steel by inducing the intergranular fatigue cracking. However, they also observed that the film-like RA located at the tip of small cracks transformed into martensite, effectively arresting crack propagation and altering the activation of slip systems. There are also other features of RA affecting its mechanical response under dynamic loading conditions. Bhattacharya et al. [78] reported a beneficial influence of mechanical twins in RA on the impact toughness of medium-Mn bainitic steel. The coherent twin boundary provides a favorable path for dislocation motion facilitating stress relaxation and blunting of the crack tip [78,80,81]. Such effect was also widely observed in austenitic high-Mn steels [82,83,84]. A schematic illustration of the fracture mechanisms in multiphase steels depending on the morphology of RA is presented in Figure 6.

The presence of RA in the microstructure may offer benefits in terms of their mechanical response under various loading conditions. The apparent contradiction regarding the influence of RA on impact strength and fracture toughness is related to the fact, that the effect of RA on the mechanical properties depends on its mechanical stability, morphology, grain size, chemical composition and location within the microstructure. RA with adequate mechanical stability, occurring as thin films between bainite/ferrite or martensite laths, may significantly improve impact strength and fracture toughness. In contrast, RA with low stability occurring in blocky form or as MA islands, particularly when is located along the PAGs, can have an unfavorable effect on mechanical properties of steel. Therefore, the contradictory observations reported in the literature can be reconciled by assuming that the effect of RA on impact strength and fracture toughness depends on several factors such as its mechanical stability, morphology (film-like or blocky) and distribution within the matrix (martensitic, bainitic or ferritic). The main challenge is to design the heat- or thermomechanical treatment conditions, which enable the formation of an adequate fraction of RA with beneficial morphology and optimal stability.

### 3.2. Medium-Mn Multiphase Steels for Forging Applications

The most promising group of multiphase steels containing RA, which show a good trade-off between mechanical properties and production costs, are medium-Mn steels. Typically, medium-manganese steels contain 0.2–0.5 wt.% C, 3–12 wt.% Mn, 0.5–1.5 wt.% Si and 0.5–1.5 wt.% Al [15,66,75]. They may also contain Mo, Cr, Ni and Cu additions and microalloying elements such as Nb, Ti and V [84,85,86,87]. Carbon and manganese contribute to the stabilization of austenite and significantly increase the hardenability of the steel, which is especially important for forgings. Increasing hardenability through the addition of Mn is a more cost-effective approach than introducing expensive Cr into the steel [88]. A reduced carbon content is beneficial in terms of machinability of forgings. The addition of Si and Al suppresses carbide formation, which has a beneficial effect on the toughness of steel [89]. This also results in a higher carbon content in RA leading to an increase in its fraction and stability. An addition of Mo enhances hardenability, hot ductility and solid solution strengthening [90]. Ni and Cu have a positive effect on the stability of RA because these elements reduce the M_s_ temperature [91,92]. Ni may form NiAl precipitates, which contribute to an increase in yield strength via precipitation strengthening. This enables an improvement in toughness through grain refinement [93]. Microadditions of Nb, Ti and V are introduced to the steel to inhibit austenite grain growth during austenitization, which enhances yield strength and toughness through the formation of complex C and N-containing compounds [94,95].

The microstructure of medium-Mn steel is composed of a ferritic, bainitic or martensitic matrix and about 10–40 vol.% of RA depending on the applied processing route. RA plays a key role in determining the mechanical properties of medium-Mn steels, under static, dynamic and cyclic loading. Under static loading, metastable RA enables a simultaneous increase in both strength and ductility of medium-Mn multiphase steels (TRIP effect). The transformation of austenite into martensite delays strain localization and necking, thereby improving fracture toughness and load-bearing capacity [96]. Under dynamic and impact loading, RA may significantly enhance impact toughness. However, its mechanical and thermal stability is crucial. RA showing stability may undergo rapid martensitic transformation, leading to local stress concentrations and therefore reduced fracture toughness.

An important aspect of medium-Mn steels containing RA is their behavior under complex stress states and the microstructural stability during long-term service. The mechanical stability of RA and its distribution within the microstructure play a key role, influencing local strain distribution and the activation of the TRIP effect under variable loading conditions. Chang et al. [97] investigated the 0.4C-7Mn-3.2Al steel under two different loading conditions, interrupting the tests after selected numbers of cycles to measure the RA content. They reported that the effectiveness of the TRIP effect depends on the number of cycles. They observed that the fraction of RA transformed into martensite increases from 6.17% after 50 cycles to 59.93% after 220 cycles and from 19.12% after 100 cycles to 43.15% after 220 cycles. In work [98], cyclic loading tests were performed on a 0.4C-7Mn-3.2Al steel. It was found that, at room temperature and at 200 °C, continuous RA transformation during cyclic loading enhances the material’s plastic deformation capacity. In contrast, at 300 °C, the stability of RA increases, making its transformation more difficult. However, systematic experimental studies on medium-Mn steels containing RA under multiaxial loading are still limited, highlighting an important direction for future research. Microstructures containing RA can achieve high strength (YS > 800 MPa and UTS > 1000 MPa) while maintaining good ductility (TEl > 20%) as well as high impact toughness (KV > 100 J) and high fatigue crack resistance [96,97,98,99]. The beneficial combination of mechanical properties and a low amount of alloying elements makes medium-Mn steels an attractive and cost-effective material for forgings. In practical applications of medium-Mn steels, particularly for forgings, careful attention should be paid to controlling the volume fraction and stability of RA through an appropriate design of the chemical composition (especially Mn, C, Al and Si) as well as heat treatment and thermomechanical processing parameters.

#### 3.2.1. Ferritic Steels Containing Retained Austenite

**Figure 7:**
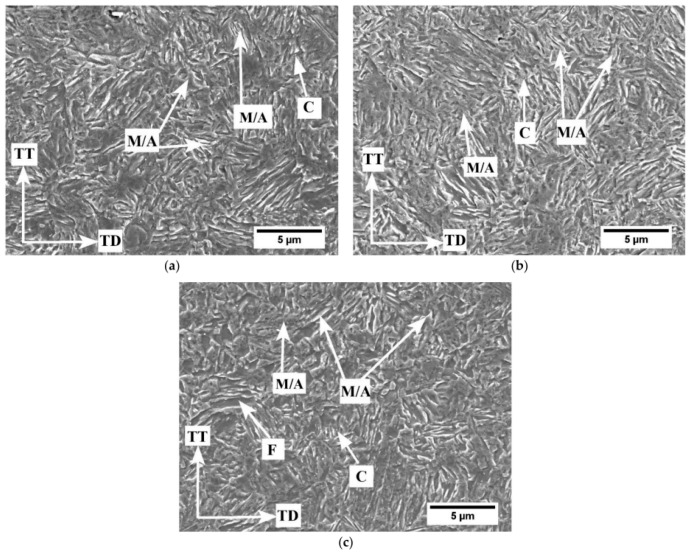
SEM micrographs of samples annealed at 675 °C for 120 s (**a**); 360 s (**b**); 600 s (**c**); C—carbide; F—ferrite; M—martensite and A—retained austenite. Reprinted from [100].

This type of steel is characterized by a dual-phase microstructure consisting of ferrite and about 20–40 vol.% of RA. The ferritic–austenitic microstructure of the 0.2C-6Mn-1.7Si-0.4Al-0.5Cr steel after intercritical annealing at 675 °C for different holding times is shown in Figure 7.

**Figure 8:**
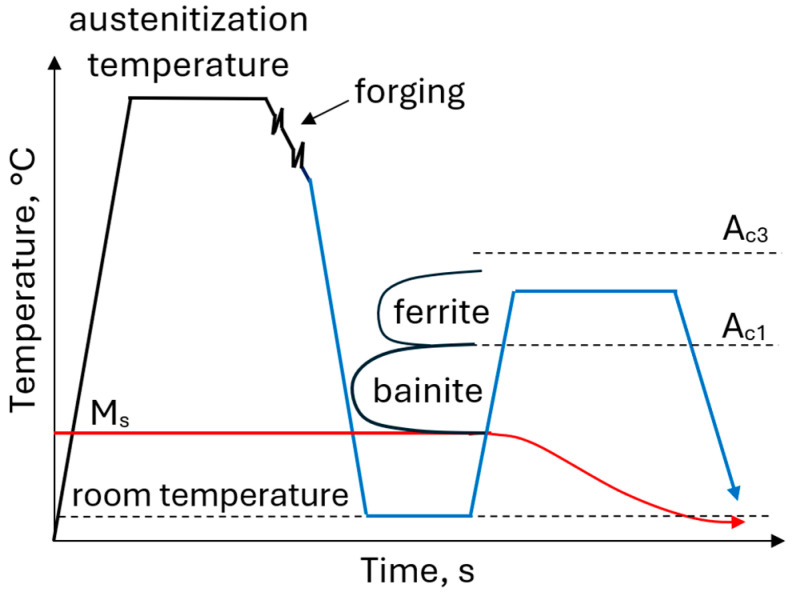
Processing schedule for forgings with a ferritic–austenitic microstructure.

The processing schedule consists of hot deformation followed by quenching to the room temperature. Then, the steel is reheated to the intercritical temperature between Ac_1_ and Ac_3_ (typically 600–700 °C) after which the steel is cooled to room temperature (Figure 8). During this process, the diffusion of C and Mn from ferrite lath contributes to the stabilization of austenite [96,101,102]. An increased manganese addition (5–12 wt.%) promotes austenite stabilization and reduces the Ac_3_ temperature, thus enabling annealing at lower temperature range [100,101,102]. This process can be easily applied in the industry for large forged components due to its relatively simple temperature control.

**Figure 9:**
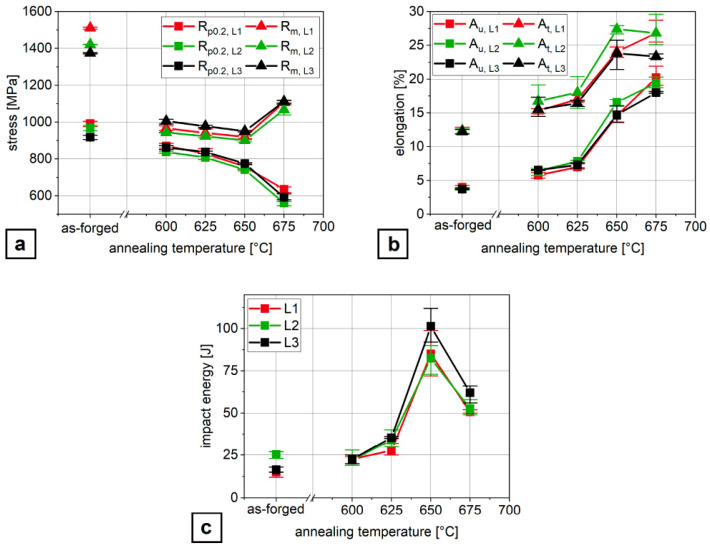
Mechanical properties of steels containing 4 wt.% Mn in the as-formed state and after intercritical annealing at different temperatures: the yield strength (YS) and ultimate tensile strength (UTS) (**a**), uniform Au and total elongation At (**b**) and the impact energy (**c**). Reprinted from [102].

The ferritic–austenitic microstructure exhibits lower strength but significantly higher ductility comparing to the as-quenched state. Due to the substantial fraction of RA, it is possible to achieve very good ductility (TEl > 20%) and impact toughness (KV > 100 J) [103]. The application of intercritical annealing after forging enables a significant increase in ductility (Figure 9b) and Charpy impact energy (Figure 9c), with a slightly lower strength (Figure 9a) compared to the as-forged state without heat treatment.

The mechanical properties of various medium-manganese steels with a ferritic–austenitic microstructure were summarized in Table 6.

The high impact toughness can be achieved through optimal design of the time-temperature parameters of intercritical annealing. On the one hand, an annealing temperature cannot be too high because it leads to the formation of high fraction of austenite, which shows low stability due to insufficient enrichment in C and Mn. On the other hand, an annealing temperature can not be too low, as a small fraction of austenite can not provide high ductility [96,103,104]. Man et al. [103] observed ductile fracture and high impact toughness (147 J) for a 0.16C-6.5Mn forged steel annealed at 600 °C, which was attributed to the high volume fraction (above 30%) and the moderate stability of RA. For annealing temperatures above 600 °C they observed a brittle, intergranular fracture and a significant decrease in impact toughness, which was attributed to the low stability of RA.

Besides the intercritical temperature, the austenitization temperature also affects the fracture behavior of medium-Mn steel. Dutta et al. [105] observed that decreasing the austenitization temperature from 1100 °C to 850 °C contributed to an increase in the strength, ductility and impact toughness of a Fe-0.06C-12Mn-3Al steel. Smaller austenite colonies exhibiting a more homogeneous microstrain distribution were formed at the lower austenitization temperature of 850 °C. This resulted in a more uniform martensitic transformation during plastic deformation, leading to the improved mechanical performance. The difference in the fracture mode of samples austenitized at 1100 °C and 850 °C were especially visible for the impact test performed at -196 °C. The sample austenitized at 1100 °C exhibited brittle intergranular fracture, while the sample austenitized at a lower temperature of 850 °C showed ductile fracture with numerous dimples.

The impact toughness of intercritically annealed samples depends also on a morphology of the RA. Han et al. [106] investigated the influence of RA morphology (lath/globular) in intercritically annealed Fe-7Mn-0.1C-0.5Si steel on its impact toughness. The steel with a lath-type microstructure showed a higher ductile-to-brittle transition temperature and lower low-temperature impact toughness compared to the steel with a microstructure composed of globular ferrite and RA grains. This behavior was attributed to the intergranular cracking in the lath-type microstructure observed along PAGs enriched with C, Mn and P. Such segregation of alloying elements at PAGs was not observed in the globular-type microstructure. The negative effect of the segregation on the grain boundaries can be reduced by the addition of molybdenum or boron [100,107]. These elements contribute to the strengthening of grain boundaries and reduce Mn segregation [108].

Due to the presence of a substantial fraction of RA (20–40 vol.%) in the microstructure of intercritically annealed steel, the microstructural features of RA have a strong influence of its fatigue behavior. Qi et al. [109] investigated the high-cycle fatigue behavior of the intercritically annealed Fe-5Mn-0.05C-0.4Cr-0.2Si-0.16Mo steel. They observed that the lath-type RA enhanced the fatigue properties of the investigated steel by delaying crack initiation and increasing resistance to crack growth through the TRIP effect. The transformation of RA into martensite within the small plastic zone relaxed the local stress concentration and increased the steel’s capacity for plastic deformation, and as a result, the initiation of microcracks was delayed [110].

#### 3.2.2. Martensitic Steels Containing Retained Austenite

**Figure 10:**
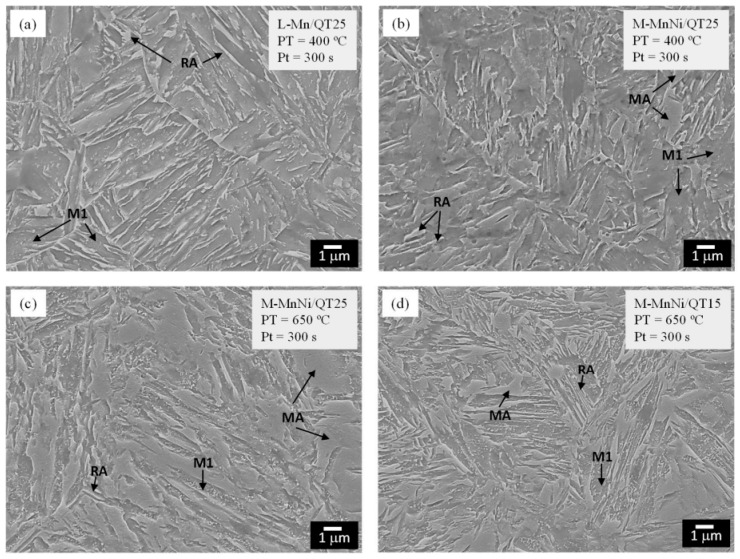
Field emission gun scanning electron microscopy (FEGSEM) micrographs corresponding to the following materials and conditions: (**a**) steel containing 2 wt.% of Mn, QT25, PT = 400 °C, and Pt = 300 s; (**b**–**d**) steel containing 5.6 wt.% of Mn, where (**b**) QT25, PT = 400 °C, Pt = 300 s, and (**c**) QT25, PT = 650 °C, and Pt = 300 s; (**d**) QT15, PT = 650 °C, and Pt = 300 s. M1—primary martensite; MA—martensite/austenite islands; RA—retained austenite. Reprinted from [112].

A microstructure consisting of martensite and RA (10–20 vol.%) can be obtained through a quenching and partitioning (Q&P) heat treatment applied after hot deformation and cooling to room temperature or through an integrated, high-efficiency thermomechanical processing [12,15,17,68,69,111]. The martensitic–austenitic microstructures of the medium-Mn steels after quenching and partitioning heat treatment performed at various partitioning temperatures are shown in Figure 10.

**Figure 11:**
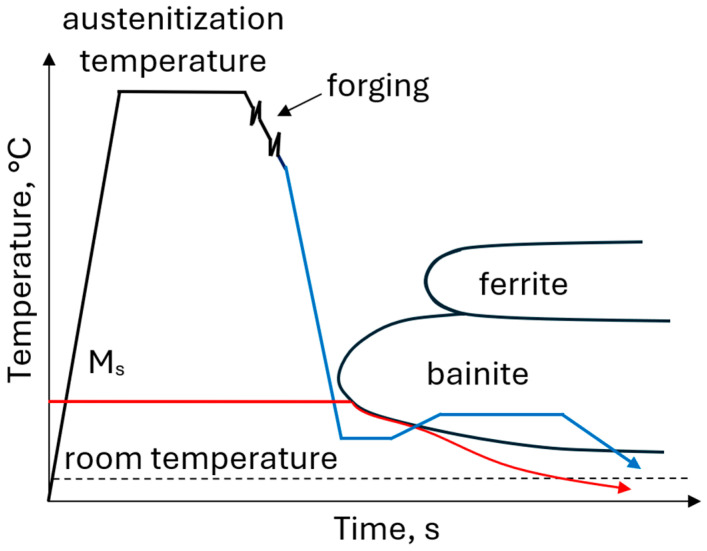
Processing schedule for forgings with a martensitic–austenitic microstructure.

The thermomechanical approach includes austenitization and hot deformation followed by direct quenching to a temperature range between martensite start (M_s_) and martensite finish (M_f_) to produce the microstructure composed of martensite and austenite. In the second stage, called partitioning, steel is reheated to the higher temperature (typically 400–450 °C) to provide conditions for carbon diffusion from supersaturated martensite enabling the stabilization of the austenite (Figure 11). An increased Mn content of 4–5 wt.% significantly reduces the M_s_ temperature and shifts the ferritic, pearlitic and bainitic transformation ranges toward longer times enabling the formation of a homogeneous martensitic–austenitic microstructure even in forgings with large cross-sections [113,114,115]. The formation of a homogeneous martensitic microstructure is possible even for very slow cooling rates of 0.05 °C/s [116].

This type of thermomechanical treatment allows the manufacturing of forgings showing very high strength (YS > 1200 MPa and UTS > 1500 MPa) with a reduced ductility (TEl 10–15%) [12,15,17,68,69]. The mechanical properties of various medium-manganese steels with a martensitic–austenitic microstructure were summarized in Table 7.

**Figure 12:**
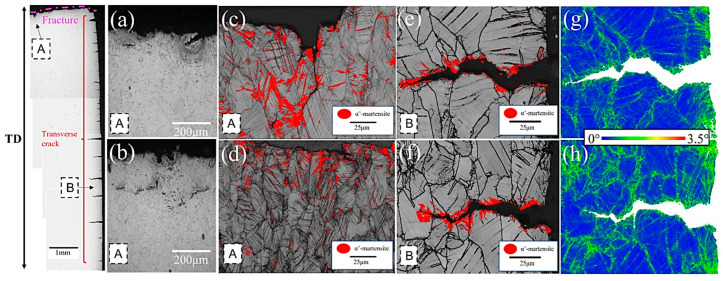
The microstructure of steel containing 5 wt.% Mn after the tensile test: fracture from the area “A” (**a**,**b**); band contrast + phase distribution map—martensite marked in red (**c**,**d**); band contrast + phase distribution map of gauge area edge from the area “B” (**e**,**f**); and KAM map (**g**,**h**). Reprinted from [119].

To ensure adequate impact toughness and crack resistance it is essential to ensure an optimal martensite–austenite ratio and appropriate conditions for austenite stabilization through carbon partitioning. Therefore, both the selection of an optimal quenching temperature and time-temperature parameters of the partitioning step are crucial. If the quenching temperature is too high a significant fraction of austenite is formed, which cannot be sufficiently enriched in carbon during the partitioning step. Yang et al. [117] reported that an increase in the quenching temperature of a Fe-0.3C-2.7Mn-1.7Si from 120 °C to 240 °C has a negative influence on its impact toughness and crack resistance because the higher fraction of low-stable blocky austenite was formed at a higher quenching temperature. The limiting of the fraction of a “fresh martensite (FM),”called also “secondary martensite (SM)” in the microstructure, especially at PAGs, is crucial. The brittle FM/SM, which formed at PAGs (from blocky austenite showing low stability) during final cooling, acts as an initiation site for crack formation [98,117,118]. Figure 12a,b shows microcracks formed in regions containing martensite. They are especially visible near the tensile fracture (Figure 12e,f). Higher kernel average misorientation (KAM) values, indicating the highest stress–strain concentrations were observed in the martensitic regions [119].

Moreover, limiting carbide precipitation at grain boundaries is important for avoiding brittle intergranular fracture because such precipitates act also as damage initiation sites [120]. The dominant, beneficial lath-type austenite morphology was observed in the sample quenched at the lower temperature of 120 °C. It was also reported that the sample quenched at this temperature showed a smaller martensite lath size and the KAM value, which resulted in more effective stress shielding effect of the martensitic matrix [117].

In addition to the features of the RA, the properties of the martensitic matrix are also of very important in terms of impact toughness and crack resistance of Q&P steel. The effect of stress/strain partitioning between the interface of two different microstructural constituents on the cohesion and consequently impact toughness of dual-phase steel was noted by the authors in [121]. Du et al. [122] reported that the nucleation areas and propagation path of microvoids and microcracks depend on the differences in deformability of martensite and austenite and on the morphology of cementite in tempered martensite. The higher difference in strength between martensite and austenite, the higher probability of crack formation at the interface between these two phases due to stress concentration and dislocation pile-up. When the difference in strength between martensite and austenite is reduced (as a result of increasing the partitioning temperature), cementite (if present) becomes the primary site for crack initiation. Another important factor affecting the impact toughness and crack resistance of Q&P steel is the dislocation mobility in martensitic laths. When the dislocation mobility is limited due to insufficient tempering, the resulting increase in flow stress reduces the impact toughness of martensitic matrix [77]. Wang et al. [77] reported that increasing the partitioning temperature of Fe-2.94Mn-0.3C-1.57Si-0.022Al steel from 400 °C to 450 °C reduced the flow stress of the martensitic matrix due to dislocation recovery, the solute carbon depletion and the transformation of transition carbides into fine cementite. The fracture mode changed from brittle intergranular in the sample partitioned at 400 °C to the ductile fracture, which was dominant in the samples partitioned at 450 °C and 500 °C.

**Table 7:** Chemical compositions and corresponding mechanical properties of medium-Mn martensitic–austenitic steels.

Chemical Composition	YS [MPa]	UTS [MPa]	TEl [%]	KV [J]	Ref.
0.3C-2.7Mn-1.7Si (quenching temperature = 20 °C)	1560	1720	8.5	11	[117]
0.3C-2.7Mn-1.7Si (quenching temperature = 120 °C)	1187	1473	21.1	17	[117]
0.3C-2.7Mn-1.7Si (quenching temperature = 240 °C)	945	1420	24.7	2.6	[117]
0.295C-1.7Si-2.9Mn-0.035Nb (at low temperature −40 °C)	1362 ± 3	1560 ± 10	16.5 ± 0.7	-	[124]
0.295C-1.7Si-2.9Mn-0.035Nb (at room temperature 20 °C)	1358 ± 4	1504 ± 8	13.4 ± 0.3	-	[124]
0.19C-1.5Si-2.9Mn-0.036Al	904	1209	15.4	-	[125]
0.23C-2.3Mn-1.5Si-12.5Cr–0.03Ti-0.05Nb (austenitization temperature = 1000 °C)	1292 ± 18	1677 ± 2	8.6 ± 0.6	53.3 ± 10.2	[126]
0.23C-2.3Mn-1.5Si-12.5Cr–0.03Ti-0.05Nb (austenitization temperature = 1020 °C)	1250 ± 28	1682 ± 4	10.2 ± 0.2	68.2 ± 6.3	[126]
0.23C-2.3Mn-1.5Si-12.5Cr–0.03Ti-0.05Nb (austenitization temperature = 1040 °C)	1258 ± 25	1739 ± 40	11.5 ± 0.7	77.2 ± 5.3	[126]
0.23C-2.3Mn-1.5Si-12.5Cr–0.03Ti-0.05Nb (austenitization temperature = 1060 °C)	1237 ± 19	1740 ± 8	10.3 ± 0.7	94.6 ± 8	[126]

#### 3.2.3. Bainitic Steels Containing Retained Austenite

**Figure 13:**
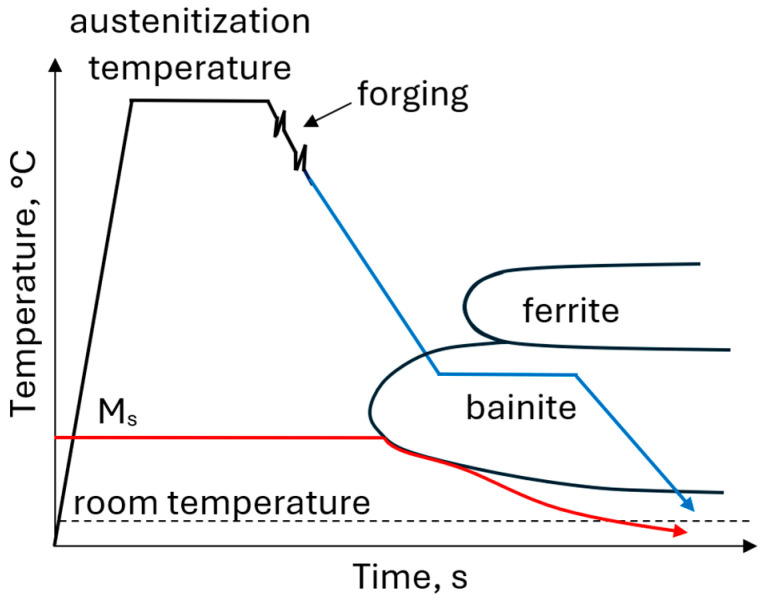
Processing schedule for forgings with a bainitic–austenitic microstructure.

**Figure 14:**
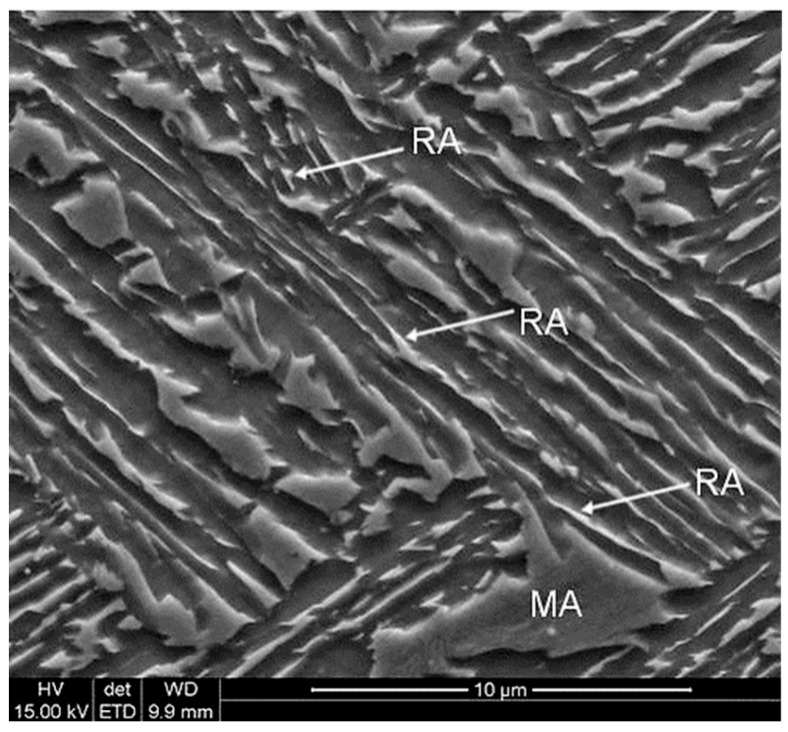
SEM micrograph of the 0.17C-3.1Mn-1.6Al-0.22Si-0.22Mo-0.04Nb steel characterized by bainitic ferrite laths containing retained austenite (RA) and martensite–austenite (MA) constituents. Reprinted from [128].

A dual-phase microstructure consisting of bainite and RA can be obtained through the thermomechanical treatment involving austenitization, hot deformation and subsequent controlled cooling to the bainitic transformation range, followed by isothermal holding typically performed at 350–450 °C (Figure 13). The bainitic–austenitic microstructure of the 0.17C-3.1Mn-1.6Al-0.22Si-0.22Mo-0.04Nb steel obtained by isothermal holding at 400 °C for 300 s is shown in Figure 14. Conventional bainitic steels contain cementite; however, in steels with RA the Si and/or Al addition suppresses cementite precipitation. As a result, carbon diffuses from the bainitic laths into the RA during the isothermal holding step leading to the stabilization at about 10–20 vol.% of this phase to the room temperature. An increased manganese content (3–5 wt.%) slows down the bainitic transformation and shifts its transformation range toward longer times. This effect is partially compensated by the Al addition, which accelerates the kinetics of the bainitic transformation. The addition of Al and Si affects also the M_s_ temperature. Increasing the Al content leads to an increase in the M_s_ temperature, whereas Si shows an opposing effect. Proper adjustment of the Al–Si ratio allows for the optimization of thermomechanical treatment and heat treatment conditions [87,123]. The fraction and stability of RA depend mainly on the time-temperature parameters of the isothermal holding step. Bainitic–austenitic steels exhibit strength and ductility levels between martensitic–austenitic and ferritic–austenitic steels (YS > 800 MPa; UTS > 1100 MPa; and TEl > 14%) [22,66,75,124,125,126,127]. Mechanical properties of various medium-Mn steels with a bainitic–austenitic microstructure are summarized in Table 8.

**Figure 15:**
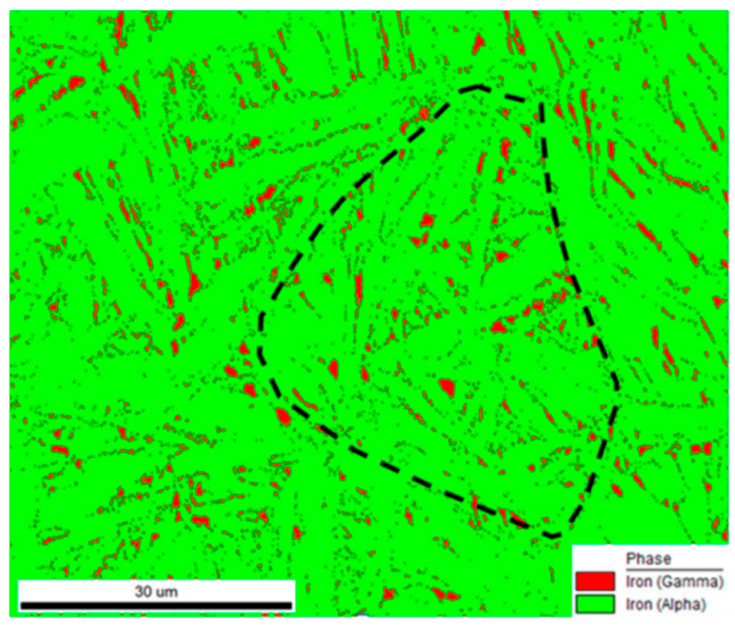
Phase distribution map of bainitic–austenitic steel. RA marked in red. The dashed line indicates prior austenite grain boundary (PAGs). Reprinted from [130].

Similar to ferritic and martensitic steels containing RA, the impact toughness and crack resistance of bainitic steel strongly depends on the morphology, fraction and stability of the RA [22]. Reduction of the fraction of structural constituents such as MA islands, which act as crack initiation sites, has a beneficial effect on the impact toughness of bainitic–austenitic steel [69]. Moreover, an improvement in impact toughness and fracture resistance can be achieved by selecting the optimal austenitization temperature. This parameter determines the PAGs and, consequently, the size of the bainite packets and blocks. Finer bainite packets and blocks lead to an increase in the fraction of high-angle grain boundaries and therefore improves the crack propagation resistance of the steel [129]. Li et al. [129] reported that reducing the austenitization temperature of the Fe-0.38C-2.19Mn-1.8Cr-1.78Si-1.3Ni-0.32Mo steel from 880 °C to 800 °C resulted in a significant reduction in the fraction of undesirable MA islands in the microstructure of bainitic–austenitic steel. Furthermore, the decrease in austenitization temperature had a positive effect on the steel’s ductility due to the grain refinement effect, which led to an increase in the stability of RA. The authors in [129] also observed that the Charpy impact energy of samples austenitized at 800 °C was significantly higher (102 J) than for samples austenitized at 880 °C (42 J). For RA to contribute to increased toughness and fracture resistance, it should exhibit a fine morphology in the form of thin laths uniformly distributed within the microstructure. The presence of RA at PAG boundaries negatively affects the toughness of steel (Figure 15).

**Table 8:** Chemical compositions and corresponding mechanical properties of medium-Mn bainitic–austenitic steels.

Chemical Composition	YS [MPa]	UTS [MPa]	TEl [%]	Hardness	KV [J]	Ref.
0.2C-1.52Si-2.98Mn-0.037Al-0.0034N	1035	1375	14.6		127	[54]
0.21C-1.5Si-4.94Mn-0.032Al-0.002N	968	1633	14.6		93	[54]
0.181C-0.97Si-2.50Mn-0.20Cr-0.096Mo-0.021Ni-0.0095Al-0.0018B-0.21Cu-0.0069N-0.032Ti (after continuous cooling)	870	1187	15.3	-	46	[64]
0.181C-0.97Si-2.50Mn-0.20Cr-0.096Mo-0.021Ni-0.0095Al-0.0018B-0.21Cu-0.0069N-0.032Ti (after isothermal holding)	898	1236	16.8	-	50	[64]
0.38C–2.19Mn–1.8Cr–1.78Si–1.3Ni–0.32Mo (austenitization temperature = 800 °C)	805	1496	22.4	525 HV	102	[129]
0.38C–2.19Mn–1.8Cr–1.78Si–1.3Ni–0.32Mo (austenitization temperature = 880 °C)	859	1757	18.6	514 HV (BF) 601 HV (MA)	42	[129]
0.23C-0.47Si-3.22Mn-1.16Al	1029 ± 22	1356 ± 9	14.7 ± 0.5	42 ± 0.2 HRC	195	[131]
0.24C-0.94Si-2.9Mn-0.72Al	1017 ± 17	1328 ± 19	14.2 ± 0.7	41 ± 0.2 HRC	190	[131]

Another important factor affecting the impact toughness and crack resistance of bainitic–austenitic steels is strain partition between individual microstructure constituents. It was reported [131] that the refinement of bainitic laths reduces the strength difference between bainite and hard MA islands, enhancing a more uniform strain distribution during deformation. This enables the reduction of the stress concentrations that occurs at the interface between bainite and MA islands. The hardness difference between bainite and MA islands lead to the inhibition of strain partitioning from bainite to MA, and therefore strain was accumulated at their interface. The MA regions act as obstacles to the transmission of plastic flow and the differences in the plasticity between the bainite and MA islands promote the nucleation of micro-void at their interface.

## 4. Summary and Conclusions

Steels intended for forging applications should exhibit high mechanical performance under static, dynamic and cyclic loading conditions. Moreover, they should show ad-equate hardenability, while maintaining good machinability. Achieving the desired properties should be possible at the lowest possible costs, which are associated with minimizing the amount of alloying additions introduced into the steel and reducing additional heat treatment operations. Meeting these requirements demands different material and technological approaches than those ones currently used in the industry. Based on the literature overview, medium-Mn multiphase steels showing high mechanical and in-use properties exceeding the conventional steels are promising materials for modern forgings. An overview of the mechanical and in-use properties of conventional and modern AHSS steels for forgings is shown in Table 9.

The mechanical and service properties of conventional and modern AHSS steels depend on their microstructural features, as summarized in Table 9. Ferritic–pearlitic steels benefit from a relatively soft ferritic matrix and lamellar pearlite, which results in relatively high fatigue strength. However, the limited fraction of high-strength phases restrict their strength. In contrast, fully martensitic microstructures exhibit high strength and hardness due to the high dislocation density but at the expense of ductility, toughness and increased alloying requirements. Furthermore, the presence of hard martensite reduces machinability. Bainitic steels offer an intermediate strength–ductility balance. Nevertheless, their industrial application in forgings is limited by difficulties in achieving microstructural homogeneity. The dual-phase steels, including ferritic–bainitic and ferritic–martensitic grades, enable a more favorable compromise between strength, ductility and fatigue performance. However, the required multistage thermomechanical treatments are associated with increased production costs, which may limit their industrial applicability. A promising alternative to conventional steel grades for forgings are modern AHSS steels containing ductile RA. This generation of multiphase steels provides a favorable balance between strength and ductility, while maintaining properties essential for forgings, such as good hardenability, high impact toughness and high fatigue strength. Moreover, the relatively low alloying content ensures economic attractiveness. Martensitic–austenitic steels are characterized by very high strength combined with slightly reduced ductility. In contrast, ferritic–austenitic steels exhibit high ductility accompanied by slightly lower strength levels. Bainitic–austenitic steels, in turn, demonstrate mechanical properties intermediate between martensitic–austenitic and ferritic–austenitic steels. Thus, medium-Mn steels provide a wide range of mechanical properties for forgings.

The most relevant findings from the literature review concerning multiphase medium-Mn steels include the following:

- **Mechanical properties under a static type of load:** The outstanding tensile properties of medium-Mn steels are attributed to the SIMT of fine, lath-type RA with optimized stability, providing an excellent strength–ductility balance. Ferritic–austenitic steels exhibit lower strength (YS > 700 MPa and UTS > 900 MPa) but higher ductility (TEl > 20%) due to a high fraction of ductile RA (20–40 vol.%). In contrast, martensitic–austenitic steels show very high strength (YS > 1200 MPa and UTS > 1500 MPa) accompanied by reduced ductility (TEl = 10–15%). Bainitic steels containing RA show intermediate strength and ductility levels between these two microstructural types (YS > 800 MPa, UTS > 1100 MPa, and TEl > 14%).
- **Mechanical properties under a dynamic type of load:** The impact toughness and crack resistance of medium-Mn steels are primarily related to the features of RA, as well as by the properties of the ferritic, martensitic or bainitic matrix. Lath-type RA with optimal stability enhances energy absorption and retards crack propagation through strain-induced martensitic transformation and crack closure mechanism. In contrast, unstable blocky RA transforming into MA islands promotes intergranular fracture along PAGs. Therefore, achieving an adequate fraction of lath-type RA with optimized stability is crucial. Additionally, stress–strain partitioning at phase interfaces influences toughness because large strength differences between the matrix and RA increase the susceptibility to interfacial cracking due to strain localization.
- **Mechanical properties under a cyclic type of load:** The fatigue behavior of multiphase steels is closely related to the characteristics of RA. MA islands and blocky RA located at PAGs negatively affect fatigue behavior by promoting intergranular crack initiation. In contrast, lath-type RA located at crack tips may transform into martensite, delaying crack initiation and retarding crack growth through the TRIP effect by relaxing local stress concentrations and enhancing plastic deformation capacity.
- **Machinability and hardenability:** The combination of low carbon (0.2–0.3 wt.%) and increased manganese (3–7 wt.%) contents enables medium-Mn steels to achieve both high hardenability and good machinability. The enhanced hardenability due to Mn allows for the air-hardening of components with various cross-sections more cost-effectively than using expensive Cr in conventional steels.
- **Production costs and environmental performance:** The use of thermomechanical processing instead of multi-step heat treatment allows for the reduction of production costs, lower energy consumption and the limitation of harmful CO_2_ emissions.

## Figures and Tables

**Table 1:** Conventional grades of steel for forgings.

Type of Microstructure	Forging Temperature [°C]	Post-Forging Cooling Conditions	Processing Method	Ref
Tempered martensite	1050–1200	Direct quenching	Water quenching directly from the finish forging temperature followed by high-temperature tempering.	[3]
Dislocated martensite	1050–1200	Direct quenching	Rapid cooling in water or other media immediately after forging, without subsequent tempering.	[12]
Ferritic–pearlitic	950–1150	Controlled, relatively slow cooling	Cooling from the finish forging temperature with a cooling rate depending on the forging cross-section size.	[24]
Bainitic	1000–1150	Controlled cooling	Cooling from the finish forging temperature with a cooling rate depending on the chemical composition of the steel.	[10]
Ferritic–bainitic and ferritic–martensitic	1000–1200	Two-stage cooling	Two-stage cooling after forging followed by low-temperature tempering (ferritic–martensitic)	[11]

**Table 2:** Chemical compositions and corresponding mechanical properties of selected commercial steels with a microstructure of tempered martensite.

Type of Steel	C	Mn	Si	Cr	Ni	Mo	YS [MPa]	UTS [MPa]	KV [J]	Ref.
30CrMnSiNi2A	0.26 ÷ 0.33	1.00 ÷ 1.30	0.90 ÷ 1.20	0.90 ÷ 1.20	1.40 ÷ 1.80	-	-	≥1600	≥60	[27]
4340	0.38 ÷ 0.43	0.60 ÷ 0.80	0.20 ÷ 0.35	0.70 ÷ 0.90	1.65 ÷ 2.00	0.20 ÷ 0.30	-	1980	20	[27]
300M	0.41 ÷ 0.46	0.65 ÷ 0.90	1.45 ÷ 1.80	0.65 ÷ 0.95	1.60 ÷ 2.00	0.30 ÷ 0.40	-	2050	24	[27]
42CrMo4	0.38 ÷ 0.45	0.60 ÷ 0.90	0.10 ÷ 0.40	0.90 ÷ 1.20	1.60 ÷ 2.00	0.15 ÷ 0.30	≥1044	≥1151	-	[28]
34CrNiMo6	0.30 ÷ 0.38	0.50 ÷ 0.80	<0.40	1.30 ÷ 1.70	1.30 ÷ 1.70	0.15 ÷ 0.30	≥1110	1000–1200	45	[29]

**Table 3:** Chemical compositions and corresponding mechanical properties of selected ferritic–pearlitic steels obtained using different cooling media.

Chemical Composition	Cooling Medium	YS [MPa]	UTS [MPa]	TEl [%]	Hardness	KV [J]	Ref.
0.35C-1.5Mn-0.6Si-0.1V	Forced air	620	-	-	270 HB	14	[38]
	Box air	660	-	-	300 HB	28	
0.35C-1.2Mn-0.7Si-0.08V-0.04Ti	Forced air	600	-	-	190 HB	46	[38]
	Box air	615	-	-	210 HB	48	
0.32C-0.79Mn-0.89Si	Normal Air	406	650	20	184 HV	107	[42]
	Oil	519	723	10	264 HV	73	
0.4C-1.21Mn-0.19Si-0.085V-0.0058N	Normal Air	525	847	23	266 HV	93	[42]
	Oil	789	1051	10	430 HV	29	
0.39C-1.27Mn-0.53Si-0.016Al-0.0048N	Sand	459	794	18	235 HV5	-	[43]
	Normal Air	490	823	17	244 HV5	-	
0.38C-1.28Mn-0.52Si-0.08V-0.007Al-0.0056N	Sand	595	890	19	273 HV5	-	[43]
	Normal Air	650	944	19	289 HV5	-	

**Table 4:** Chemical compositions and corresponding mechanical properties of bainitic steels.

Chemical Composition	Microstructure	YS [MPa]	UTS [MPa]	TEl [%]	Hardness	KV [J]	Ref.
0.183C-0.97Si-2.39Mn-0.02Al-0.25Cr-0.09Mo-0.019Ni-0.0018B-0.21Cu-0.033Ti	Bainitic	734	1062	-	-		[48]
0.34C-0.19Si-1.61Mn-0.80Cr-0.30Mo-0.027Al-0.002Nb-0.0036B-0.007N	Globular bainite grains, lamelas, grain size: 10–30 μm	732 ± 22	1114 ± 28	11.6	332 HV30	6	[53]
0.20C-0.26Si-(1.50–2.10)Mn-(1.40–1.85)Cr-0.40Mo-0.023Al-0.060Nb-0.003B-0.019N	Globular bainite grains, grain size: 10 μm	721 ± 11	1055 ± 11	14.9	343 HV30	15	[53]

**Table 5:** Mechanical properties of dual-phase ferritic–bainitic/ferritic–martensitic and single-phase bainitic/martensitic steels.

Chemical Composition	Type of Microstructure	YS [MPa]	UTS [MPa]	TEl [%]	Hardness [HRC]	KV [J]	Ref.
0.4C-0.29Si-0.62Mn-0.73Cr-1.77Ni-0.21Mo	Bainitic	1494	1706	12.5	48	19.7	[61]
0.4C-0.29Si-0.62Mn-0.73Cr-1.77Ni-0.21Mo	Ferritic–bainitic	1244	1424	13.8	41	36.5	[61]
0.4C-0.29Si-0.62Mn-0.73Cr-1.77Ni-0.21Mo	Ferritic–martensitic	1914	2190	8.1	45	10.4	[61]
0.4C-0.29Si-0.62Mn-0.73Cr-1.77Ni-0.21Mo	Martensitic	2000	2200	5.1	56	6.0	[61]
0.27C-0.71Mn-1.08Cr-0.29Si-0.24Mo-0.25Ni-0.037V-0.03Cu	24% martensitic, 76% bainitic	572	730	21.5	-	78	[62]
0.27C-0.71Mn-1.08Cr-0.29Si-0.24Mo-0.25Ni-0.037V-0.03Cu	45% martensitic, 55% bainitic	594	753	22.5	-	88	[62]
0.27C-0.71Mn-1.08Cr-0.29Si-0.24Mo-0.25Ni-0.037V-0.03Cu	75% martensitic, 25% bainitic	624	774	22.0	-	82	[62]

**Table 6:** Chemical compositions and corresponding mechanical properties of medium-Mn ferritic–austenitic steels.

Chemical Composition	YS [MPa]	UTS [MPa]	TEl [%]	Hardness	KV [J]	Ref.
0.16C-4.7Mn-1.6Al-0.2Si-0.2Mo (IA temperature = 680 °C; IA time = 1 min)	859 ± 16	938 ± 18	18.0± 0.6	318 ± 10 HV1	-	[96]
0.16C-4.7Mn-1.6Al-0.2Si-0.2Mo (IA temperature = 680 °C; IA time = 60 min)	654	918	28.3	295 HV1	-	[96]
0.16C-4.7Mn-1.6Al-0.2Si-0.2Mo (IA temperature = 680 °C; IA time = 300 min)	591 ± 11	890 ± 20	26.5 ± 0.8	279 ± 8 HV1	-	[96]
0.17C-3.99Mn-0.025Al-0.50Si-0.02Mo-0.02Ti-0.033Nb-0.0057B-0.01N (IA temperature = 650 °C)	742	900	27.4	294 HV10	82	[102]
0.17C-3.99Mn-0.025Al-0.50Si-0.02Mo-0.02Ti-0.033Nb-0.0057B-0.01N (IA temperature = 675 °C)	561	1067	26.8	288 HV10	53	[102]
0.15C-4.02Mn-0.027Al-0.49Si-0.20Mo-0.003Ti-0.035Nb-0.0005B-0.01N (IA temperature = 650 °C)	775	951	23.8	312 HV10	101	[102]
0.15C-4.02Mn-0.027Al-0.49Si-0.20Mo-0.003Ti-0.035Nb-0.0005B-0.01N (IA temperature = 675 °C)	592	1112	23.4	301 HV10	62	[102]
0.16C-6.5Mn-0.02Al-0.15Si-0.02Mo-0.015Cr-0.03Ni-0.0045Co-0.017Cu-0.0008Nb-0.0017Ti-0.0032V-0.004N (IA temperature = 500 °C)	-	-	-	-	10	[103]
0.16C-6.5Mn-0.02Al-0.15Si-0.02Mo-0.015Cr-0.03Ni-0.0045Co-0.017Cu-0.0008Nb-0.0017Ti-0.0032V-0.004N (IA temperature = 600 °C)	-	-	-	-	147	[103]
0.16C-6.5Mn-0.02Al-0.15Si-0.02Mo-0.015Cr-0.03Ni-0.0045Co-0.017Cu-0.0008Nb-0.0017Ti-0.0032V-0.004N (IA temperature = 650 °C)	-	-	-	-	41	[103]

**Table 9:** Overview of mechanical and in-use properties of conventional and modern steels for forgings, evaluated on a relative scale (low, medium and high).

		Conventional Steels					Advanced High-Strength Steels		
Properties		Tempered Martensite	Dislocated Martensite	Ferritic–Pearlitic	Bainitic	Ferritic–Bainitic/ Ferritic– Martensitic	Ferritic–Austenitic	Bainitic– Austenitic	Martensitic-Austenitic
	Type of Steels								
Alloying		high	high	medium	medium	medium	medium	medium	medium
Machinability		medium	low	low	medium	medium	medium	medium	medium
Hardenability		high	high	low	medium	medium	high	high	high
Ultimate tensile strength		high	high	low	medium	medium	medium	high	high
Yield strength		high	high	medium	medium	medium	medium	high	high
Total elongation		medium	low	medium	medium	medium	high	medium	low
Hardness		high	high	medium	medium	medium	medium	high	high
Impact toughness		medium	medium	low	medium	medium	high	medium	medium
Fatigue strength		high	low	high	medium	medium	high	high	high
Production costs		high	medium	medium	medium	high	medium	low	low
Environmental performance		low	medium	medium	medium	medium	high	high	high

## Data Availability

No new data were created or analyzed in this study. Data sharing is not applicable to this article.

## References

[1] Lee Y.K., Han J. (2015). Current Opinion in Medium Manganese Steel. Mater. Sci. Technol..

[2] Sugimoto K., Hojo T., Srivastava A.K. (2019). Low and Medium Carbon Advanced High-Strength Forging Steels for Automotive Applications. Metals.

[3] Hawryluk M., Gronostajski Z., Zwierzchowski M., Jabłoński P., Barełkowski A., Krawczyk J., Jaśkiewicz K., Rychlik M. (2020). Application of a Prototype Thermoplastic Treatment Line in Order to Design a Thermal Treatment Process of Forgings with the Use of the Heat from the Forging Process. Materials.

[4] Sugimoto K., Hojo T., Mizuno Y. (2017). Torsional Fatigue Strength of Newly Developed Case Hardening TRIP-Aided Steel. Metals.

[5] Gao G., Zhang B., Cheng C., Zhao P., Zhang H., Bai B. (2016). Very high Cycle Fatigue Behaviors of Bainite/Martensite Multiphase Steel Treated by Quenching-Partitioning-Tempering Process. Int. J. Fatigue.

[6] Skubisz P., Adrian H., Sińczak J. (2011). Controlled Cooling of Drop Forged Microalloyed-Steel Automotive Crankshaft. Arch. Metall. Mater..

[7] Schmiedl T., Gramlich A., Schönborn S., Melz T. (2020). Behavior of Forging Steels under Cyclic Loading—The Benefit of Air-Hardening Martensites. Steel Res. Int..

[8] Raedt H.W., Wilke F., Ernst C.S. (2014). The Lightweight Forging Initiative Automotive Lightweight Design Potential with Forging. ATZ Worldw..

[9] Gramlich A., Helbig C., Schmidt M., Hagedorn W. (2024). A Comprehensive Design Approach to Increase the Performance of Steels Under Minimal Costs and Environmental Impacts. Sustain. Mater. Technol..

[10] Keul C., Wirths V., Bleck W. (2012). New Bainitic Steel for Forgings. Arch. Civ. Mech. Eng..

[11] Sankaran S., Subramanya V., Padmanabhan K.A. (2003). Low Cycle Fatigue Behavior of a Multiphase Medium Steel: Comparison Between Ferrite-Pearlite and Quenched and Tempered Microstructures. Mater. Sci. Eng. A.

[12] Song C., Zhang Z., Wu W., Wang H., Sun Z., Yang Y., He W., Xu J., Xia Y., Yin W. (2023). Effect of Si on the Dislocation State Within Martensite of Ultra-High Strength Hot-Rolled Medium Mn Steel with Good Ductility. Mater. Sci. Eng. A.

[13] Qian L., Zhou Q., Zhang F., Meng J., Zhang M., Tian Y. (2012). Microstructure and Mechanical Properties of a Low Carbon Carbide-free Bainitic Steel co-Alloyed with Al and Si. Mater. Des..

[14] Li Z.C., Ding H., Misra R.D.K., Cai Z.H. (2017). Microstructure-Mechanical Property Relationship and Austenite Stability in Medium-Mn TRIP Steels: The Effect of Austenite-Reverted Transformation and Quenching-Tempering Treatments. Mater. Sci. Eng. A.

[15] Mishra G., Chandan A.K., Kundu S. (2017). Hot Rolled and Cold Rolled Medium Manganese Steel: Mechanical Properties and Microstructure. Mater. Sci. Eng. A.

[16] Bleck W. Using the TRIP effect—The Dawn of a Promising Group of Cold Formable Steels. Proceedings of the International Conference on TRIP-Aided High-Strength Ferrous Alloys.

[17] Ayenampudi S., Celada-Casero C., Arechabaleta Z., Arribas M., Arlazarov A., Sietsma J., Santofimia M.J. (2021). Microstructural Impact of Si and Ni During High Temperature Quenching and Partitioning Process in Medium-Mn Steels. Metall. Mater. Trans. A.

[18] Guo H., Zhou P., Zhao A., Zhi C., Ding R., Wang J. (2017). Effects of Mn and Cr Contents on Microstructures and Mechanical Properties of Low Temperature Bainitic Steel. J. Iron Steel Res. Int..

[19] Tao R., Zeng Q., Chai X.S., Yuan L.J., Wen P.C., Li D. (2022). The Relationship Between Austenite Morphology and Continuous TRIP Effects in Forged 4Mn Steel. Mater. Charact..

[20] Opiela M., Grajcar A. (2012). Hot Deformation Behavior and Softening Kinetics of Ti-V-B Microalloyed Steels. Arch. Civ. Mech. Eng..

[21] Sugimoto K., Hojo T., Srivastava A. (2018). An Overview of Fatigue Strength of Case-Hardening TRIP-Aided Martensitic Steels. Metals.

[22] Sugimoto K., Sato S., Kobayashi J., Srivastava A. (2019). Effects of Cr and Mo on Mechanical Properties of Hot-Forged Medium Carbon TRIP-Aided Bainitic Ferrite Steels. Metals.

[23] Sugimoto K., Sato S., Arai G. The Effects of Hot-forging on Mechanical Properties of Ultra High-strength TRIP-aided Steels. Proceedings of the International Steel Technologies Symposium.

[24] Tinius C.H., Mrdjenovich R. Forged microalloyed steel crankshafts for automotive engines. Proceedings of the Fundamentals and Applications of Microalloying Forging Steels, International Symposium on Microalloyed Bar and Forging Steels, 2.

[25] Ghosh A., Chatterjee S., Mishra B. (2003). Influence of Thermo-Mechanical Processing and Different Post-cooling Techniques on Structure and Properties of an Ultralow Carbon Cu Bearing HSLA Forging. Mater. Sci. Eng. A.

[26] Gronostajski Z., Hawryluk M. (2008). The Main Aspects of Precision Forging. Arch. Civ. Mech. Eng..

[27] Zhao Y.J., Ren X.P., Hu Z.L., Xiong Z.P., Zeng J.M., Hou B.Y. (2018). Effect of Tempering on Microstructure and Mechanical Properties of 3Mn-Si-Ni Martensitic Steel. Mater. Sci. Eng. A.

[28] Ulrich C., Günther S., Becker N., Schubert V., Vetter B., Leyens C., Schlecht B. (2025). High Strength Tempered 42CrMo4 for Shafts in Drive Technology. Forsch. Im Ingenieurwesen.

[29] Janeková M., Koštialiková D., Dubec A., Burget M., Pešlová F. (2018). The Heat Treatment Impact on Material Properties of 34CrNiMo6 Steel. Manuf. Technol..

[30] Zhu C., Xiong X.Y., Cerezoa A., Hardwickea R., Krauss G., Smith G.D.W. (2007). Three-Dimensional Atom Probe Characterization of Alloy Element Partitioning in Cementite During Tempering of Alloy Steel. Ultramicroscopy.

[31] Krauss G. (2012). Tempering of Martensite in Carbon Steels. Phase Transformations in Steels: Diffusionless Transformations, High Strength Steels, Modelling and Advanced Analytical Techniques.

[32] Ren Q., Kou Z., Wu J., Hou T., Xu P. (2023). Effect of TemperingTemperature on Microstructure andMechanical Properties of 35CrMoSteel. Metals.

[33] Qi L., Khachaturyan A.G., Morris J.W. (2014). The Microstructure of Dislocated Martensitic Steel: Theory. Acta Mater..

[34] Kinney C.C., Pytlewski K.R., Khachaturyan A.G., Morris J.W. (2014). The Microstructure of Lath Martensite in Quenched 9Ni Steel. Acta Mater..

[35] Hem B.B., Huangm M.X. (2015). Revealing the Intrinsic Nanohardness of Lath Martensite in Low Carbon Steel. Metall. Mater. Trans. A.

[36] Matlock D., Speer J. (2009). Microalloying Concepts and Application in Long Products. Mater. Sci. Technol..

[37] Ollilainen V., Kasprzak W., Holappa L. (2003). The Effect of Silicon, Vanadium and Nitrogen on the Microstructure and Hardness of Air Cooled Medium Carbon Low Alloy Steels. J. Mater. Process. Technol..

[38] Jahazi M., Eghbali B. (2001). The Influence of Hot Forging Conditions on the Microstructure and Mechanical Properties of Two Microalloyed Steels. J. Mater. Process. Technol..

[39] Opiela M., Grajcar A. (2012). Elaboration of Forging Conditions on the Basis of the Precipitation Analysis of MX-Type Phases in Microalloyed Steels. Arch. Civ. Mech. Eng..

[40] Opiela M. (2014). Effect of Thermomechanical Processing on the Microstructure and Mechanical Properties of Nb-Ti-V Microalloyed Steel. J. Mater. Eng. Perform..

[41] Rasouli D., Khameneh A.S., Akbarzadeh A., Daneshi G.H. (2008). Effect of Cooling Rate on the Microstructure and Mechanical Properties of Microalloyed Forging Steel. J. Mater. Process. Technol..

[42] Mukerjee D., Ohdar R.K., Talukdar P., Equbal M. (2017). Hot Forging Behaviour of Medium Carbon and Microalloyed Steel: A Comparative Study. Int. J. Microstruct. Mater. Prop..

[43] Gündüz S., Çapar A. (2006). Influence of Forging and Cooling Rate on Microstructure and Properties of Medium Carbon Microalloy Forging Steel. J. Mater. Sci..

[44] Balart M.J., Davis C.L., Strangwood M. (2002). Fracture Behavior in Medium-Carbon Ti-V-N and V-N Microalloyed Ferritic-Pearlitic and Bainitic Forging Steels with Enhanced Machinability. Mater. Sci. Eng. A.

[45] Adrian H., Głowacz E. (2010). The Effect of Nitrogen and Microalloying Elements (V and V+Al) on Austenite Grain Growth of 40Cr8 Steel. Arch. Metall. Mater..

[46] Matlock D.K., Krauss G., Speer J.G. (2001). Microstructures and Properties of Direct-Cooled Microalloying Forging Steels. J. Mater. Process. Technol..

[47] Soliman M., Palkowski H. (2016). Development of the Low Temperature Bainite. Arch. Civ. Mech. Eng..

[48] Elek L., Wagener R., Kaufmann H., Wirths V., Melz T. (2015). New Bainitic Steel for Cyclic Loaded Safety Parts with Improved Cyclic Material Behaviour. Procedia Eng..

[49] De Figueiredo Silveira A.C., Bevilaqua W.L., Dias V.W., de Castro P.J., Epp J., Da Silva Rocha A. (2020). Influence of Hot Forging Parameters on a Low Carbon Continuous Cooling Bainitic Steel Microstructure. Metals.

[50] Zhao H., Wynne B.P., Palmiere E.J. (2017). Effect of Austenite Grain Size on the Bainitic Ferrite Morphology and Grain Refinement of a Pipeline Steel After Continuous Cooling. Mater. Charact..

[51] Müller M., Britz D., Ulrich L., Staudt T., Mücklich F. (2020). Classification of Bainitic Structures Using Textural Parameters and Machine Learning Techniques. Metals.

[52] Zajac S., Schwinn V., Tacke K.H. (2005). Characterisation and Quantification of Complex Bainitic Microstructures in High and Ultra-High Strength Linepipe Steels. Mater. Sci. Forum.

[53] Gramlich A., Lange R., Zitz U., Büßenschütt K. (2022). Air-Hardening Die-Forged Con-Rods—Achievable Mechanical Properties of Bainitic and Martensitic Concepts. Metals.

[54] Sugimoto K., Tanino H., Kobayashi J. (2015). Impact Toughness of Medium-Mn Transformation-Induced Plasticity-Aided Steels. Steel Res. Int..

[55] Caballero F.G., Bhadeshia H.K.D.H., Mawella K.J.A., Jones D.G., Brown P. (2002). Very Strong Low-Temperature Bainite. Mater. Sci. Technol..

[56] Caballero F.G., Bhadeshia H.K.D.H. (2004). Very Strong Bainite. Curr. Opin. Solid State Mater. Sci..

[57] Królicka A., Żak A., Kuziak R., Radwański K., Ambroziak A. (2021). Decomposition Mechanisms of Continuously Cooled Bainitic Rail in the Critical Heat-Affected Zone of Flash-Butt Welded Joints. Mater. Sci. Pol..

[58] Lisiecka-Graca P., Lisiecki Ł., Zyguła K., Wojtaszek M. (2024). Evaluation of Cracking Risk of 80MnSi8-6 Nanobainitic Steel During Hot Forging in the Range of Lower Temperature Limits. Mater. Sci. Pol..

[59] Marcisz J., Garbarz B., Janik A., Zalecki W. (2021). Controlling the Content and Morphology of Phase Constituents in Nanobainitic Steel Containing 0.6%C to Obtain the Required Ratio of Strength to Plasticity. Metals.

[60] González-Baquet I., Kaspar R., Richter J., Nußbaum G., Köthe A. (1997). Influence of Microalloying on the Mechanical Properties of Medium Carbon Forging Steels After a Newly Designed Post Forging Treatment. Steel Res..

[61] Saeidi N., Ekrami A. (2009). Comparison of Mechanical Properties of Martensite/Ferrite and Bainite/Ferrite Dual Phase 4340 Steels. Mater. Sci. Eng. A.

[62] Zhang Y., Cao Y., Huang G., Wang Y., Li Q., He J. (2023). Influence of Martensite/Bainite Dual Phase-Content on the Mechanical Properties of EA4T High-Speed Axle Steel. Materials.

[63] Sugimoto K., Hojo T., Kobayashi J. (2017). Critical Assessment of TRIP-aided Bainitic Ferrite Steels. Mater. Sci. Tech..

[64] Wirths W., Wagener R., Bleck W., Melz T. (2014). Bainitic Forging Steels for Cyclic Loading. Adv. Mater. Res..

[65] Kuziak R., Kawalla R., Waengler S. (2008). Advanced High Strength Steels for Automotive Industry. Arch. Civ. Mech. Eng..

[66] Grajcar A., Skrzypczyk P., Kozłowska A. (2018). Effects of Temperature and Time of Isothermal Holding on Retained Austenite Stability in Medium-Mn Steels. Appl. Sci..

[67] Guo Q., Hu B., Luo H. (2022). Mechanism and Application of Reverse Austenitic Transformation in Medium Mn Steels: A Systematic Review. Steel Res. Int..

[68] Finfrock C.B., Clarke A.J., Thomas G.A., Clarke K.D. (2020). Austenite Stability and Strain Hardening in C-Mn-Si Quenching and Partitioning Steels. Metall. Mater. Trans. A.

[69] Gulbay O., Gramlich A., Krupp U. (2025). Impact Toughness and Fatigue Crack Propagation in Carbide-Free Bainite: The Adverse Role of Retained Austenite and Martensite-Austenite Islands. Fatigue Fract. Eng. Mater. Struct..

[70] Xia P., Sabirov I., Molina-Aldareguia J., Verleysen P., Petrov R. (2018). Mechanical Behavior and Microstructure Evolution of a Quenched and Partitioned Steel During Drop Weight Impact and Punch Testing. Mater. Sci. Eng. A.

[71] Yu L., Gu X., Qian L., Jiang P., Wang W., Yu M. (2021). Application of tailor rolled blanks in optimum design of pure electric vehicle crashworthiness and lightweight. Thin-Walled Struct..

[72] Hidalgo J., Findley K.O., Santofimia M.J. (2017). Thermal and Mechanical Stability of Retained Austenite Surrounded by Martensite with Different Degrees of Tempering. Mater. Sci. Eng. A.

[73] Kim H., Lee J., Barlat F., Kim D., Lee M.G. (2015). Experiment and Modeling to Investigate the Effect of Stress State, Strain and Temperature on Martensitic Phase Transformation in TRIP-Assisted Steel. Acta Mater..

[74] Rong T., Lin L., De Cooman B.C., Chen W.X., Peng S. (2006). Effect of Temperature and Strain Rate on Dynamic Properties of Low Silicon TRIP Steel. J. Iron Steel. Res. Int..

[75] Kozłowska A., Grajcar A., Janik A., Radwański K., Krupp U., Matus K., Morawiec M. (2021). Mechanical and Thermal Stability of Retained Austenite in Plastically Deformed Bainite-Based TRIP-Aided Medium-Mn Steels. Arch. Civ. Mech. Eng..

[76] De Knijf D., Petrov R., Föjer C., Kestens L.A.I. (2014). Effect off Fresh Martensite on the Stability of Retained Austenite in Quenching and Partitioning Steel. Mater. Sci. Eng. A.

[77] Wang Z., Huang M.X. (2020). Optimising the Strength-Ductility-Toughness Combination in Ultra-High Strength Quenching and Partitioning Steels by Tailoring Martensite Matrix and Retained Austenite. Int. J. Plast..

[78] Bhattacharya A., Biswal S., Barik R.K., Mahato B., Ghosh M., Mitra R., Chakrabarti D. (2024). Comparative Interplay of C and Mn on Austenite Stabilization and Low Temperature Impact Toughness of Low C Medium Mn Steels. Mater. Charact..

[79] Gao G., Liu R., Wang K., Gui X., Misra R., Bai B. (2020). Role of Retained Austenite with Different Morphologies on Sub-Surface Fatigue Crack Initiation in Advanced Bainitic Steels. Scr. Mater..

[80] Huo C.Y., Gao H.L. (2005). Strain-Induced Martensitic Transformation in Fatigue Crack Tip Zone for a High Strength Steel. Mater. Charact..

[81] Seita M., Hanson J.P., Gradecak S., Demkowicz M.J. (2015). The Dual Role of Coherent Twin Boundaries in Hydrogen Embrittlement. Nat. Commun..

[82] Kuan Ren J., Yuan Chen Q., Chen J., Yu Liu Z. (2020). Enhancing Strength and Cryogenic Toughness of High Manganese TWIP Steel Plate by Double Strengthened Structure Design. Mater. Sci. Eng. A.

[83] Chen J., Kuan Ren J., Yu Liu Z., Dong Wang G. (2018). Interpretation of Significant Decrease in Cryogenic-Temperature Charpy Impact Toughness in a High Manganese Steel. Mater. Sci. Eng. A.

[84] Skowronek A., Hailu Kori T., Gramlich A., Gulbay O., Krupp U., Grajcar A. (2025). Thermodynamic-Kinetic and Dilatometric Characterization of the Influence of Mo and Cu Additions on Phase Transition Behavior in High-Strength Medium-Mn Steels. J. Therm. Anal. Calorim..

[85] Chen J., Dong F.T., Liu Z.Y., Wang G.D. (2021). Grain Size Dependence of Twinning Behaviors and Resultant Cryogenic Impact Toughness in High Manganese Austenitic Steel. J. Mater. Res. Technol..

[86] Grajcar A., Skrzypczyk P., Kuziak R., Gołombek K. (2014). Effect of Finishing Hot-Working Temperature on Microstructure of Thermomechanically Processed Mn–Al Multiphase Steels. Steel Res. Int..

[87] Wojtacha A., Kozłowska A., Morawiec M., Opiela M. (2025). Thermodynamic Prediction and Experimental Verification of Phase Transformation Kinetics in 3Mn Steel with Ti and V Microadditions. J. Therm. Anal. Calorim..

[88] Wang L., Liang Y., Zhao F., Xu F., Lei L., Long S., Yang M., Jiang Y. (2024). Achieving High Tensile Properties and Impact Toughness in Ultrahigh Strength Lean Alloy Steel by Quenching and Partitioning Treatment. Arch. Civ. Mech. Eng..

[89] Kaar S., Krizan D., Schneider R., Sommitsch C. (2020). Impact of Si and Al on Microstructural Evolution and Mechanical Properties of Lean Medium Manganese Quenching and Partitioning Steels. Steel Res. Int..

[90] Zvavamwe F., Pasco J., Paek M.K., Aranas C. (2025). Influence of Molybdenum Additions on the Microstructural Properties of Medium-Mn Steels. J. Mater. Res. Technol..

[91] Ma J., Song Y., Jiang H., Rong L. (2022). Effect of Cu on the Microstructure and Mechanical Properties of a Low-Carbon Martensitic Stainless Steel. Materials.

[92] Del Molino L., Arribas Telleria M., Gilliams C., Arlazarov A., Gonzalez J.G., De Moor E., Speer J.G. (2022). Influence of Ni and Process Parameters in Medium Mn Steels Heat Treated by High Partitioning Temperature Q&P Cycles. Metall. Mater. Trans. A.

[93] Królicka A., Caballero F.G. (2025). B2-strengthened Fe-Mn-Al-C-Ni Steels as a Promising Environmentally Friendly Structural Material: Review and Perspectives. Crit. Rev. Solid State Mater. Sci..

[94] Han Y., Shi J., Xu L., Cao W.Q., Dong H. (2012). Effects of Ti Addition and Reheating Quenching on Grain Refinement and Mechanical Properties in Low Carbon Medium Manganese Martensitic Steel. Mater. Des..

[95] Vercruysse F., Celada-Casero C., Linke B.M., Verleysen P., Petrov R.H. (2021). The Effect of Nb on the Strain Rate and Temperature Dependent Behaviour of Quenching & Partitioning Steels. Mater. Sci. Eng. A.

[96] Skowronek A., Grajcar A., Garcia-Mateo C., Jimenez J.A., Petrov R.H. (2023). Time-Dependent Evolution of Volume Fraction and Stability of Retained Austenite in a Hot-Rolled and Intercritically Annealed Al-Alloyed Medium-Mn Steel. Metall. Mater. Trans. A.

[97] Chang S., Zhu Z., Huang X., Zhang J., Kang G. (2024). Effect of Martensitic Transformation on Ratchetting in Medium-Manganese Steel: Experiment and Homogenized Constitutive Model. Int. J. Fatigue.

[98] Zhang J., Song Z., Wu Z., Huang X., Kan Q. (2025). Cyclic Deformation Behavior of Medium-Manganese Transformation-Induced Plasticity Steel at Elevated Temperatures: Mechanical Tests and Microstructural Characterization. Int. J. Fatigue.

[99] Kwok T.W.J., Dye D. (2023). A Review of the Processing, Microstructure and Property Relationships in Medium Mn Steels. Int. Mater. Rev..

[100] Bhadhon K.M.H., Wang X., McNally E.A., McDermid J.R. (2022). Effect of Intercritical Annealing Parameters and Starting Microstructure on the Microstructural Evolution and Mechanical Properties of a Medium-Mn Third Generation Advanced High Strength Steel. Metals.

[101] Gramlich A., Black W. (2021). Tempering and Intercritical Annealing of Air-Hardening 4 wt.% Medium Manganese Steels. Steel Res. Int..

[102] Gramlich A., Emmrich E., Bleck W. (2019). Austenite Reversion Tempering-Annealing of 4 wt.% Manganese Steels for Automotive Forging Application. Metals.

[103] Man T., Wang J., Zhao H., Dong H. (2024). Impact Toughness Dependent on Annealing Temperatures in 0.16C-6.5Mn Forged Steel for Flywheel Rotors. Metals.

[104] Skowronek A., Grajcar A., Kozłowska A., Janik A., Morawiec M., Petrov R.H. (2022). Temperature-Dependent Microstructural Evolution of Al-Rich Medium-Mn Steel During Intercritical Annealing. Metall. Mater. Trans. A.

[105] Dutta A., Park T.M., Nam J.H., Lee S.I., Hwang B., Choi W.S., Sandlobes S., Ponge D., Han J. (2021). Enhancement of the Tensile Properties and Impact Toughness of a Medium-Mn Steel Through the Homogeneous Microstrain Distribution. Mater. Charact..

[106] Han J., Kwiatkowski da Silva A., Ponge D., Raabe D., Lee S.M., Lee Y.K., Lee S.I., Hwang B. (2017). The Effects of Prior Austenite Grain Boundaries and Microstructural Morphology on the Impact Toughness of Intercritically Annealed Medium Mn Steel. Acta Mater..

[107] Song S.H., Faulkner R.G., Flewitt P.E.J. (2000). Quenching and Tempering-Induced Molybdenum Segregation to Grain Boundaries in a 2.25Cr–1Mo Steel. Mater. Sci. Eng. A.

[108] Kuzmina M., Ponge D., Raabe D. (2015). Grain Boundary Segregation Engineering and Austenite Reversion Turn Embrittlement Into Toughness: Example of a 9 wt.% Medium Mn Steel. Acta Mater..

[109] Qi X.Y., Du L.X., Hu J., Misra R.D.K. (2018). High-Cycle Fatigue Behavior of Low-C Medium-Mn High Strength Steel with Austenite-Martensite Submicron-Sized Lath-Like Structure. Mater. Sci. Eng. A.

[110] Song S.M., Sugimoto K., Kandaka S., Futamura A., Kobayashi M., Masuda S. (2003). Effects of Prestraining on High-Cycle Fatigue Strength of High-Strength Low Alloy TRIP-Aided Steels. J. Soc. Mater. Sci. Jpn..

[111] Kozłowska A., Skowronek A., Opara J., Matus K., Nuckowski P.M. (2025). Tailoring Thermal Stability of Retained Austenite in Thermomechanically Processed Medium-Mn Steel via Quenching and Partitioning Process. Arch. Civ. Mech. Eng..

[112] Arribas M., Del Molino E., Gutiérrez T., Arlazarov A., Martin D., De Caro D., Ayenampudi S., Santofimia M.J. (2022). Characterization of a Medium Mn-Ni Steel Q&P Treated by a High Partitioning Temperature Cycle. Metals.

[113] Kim J.C., Han D.W., Baik S.H., Lee Y.K. (2004). Effects of Alloying Elements on Martensitic Transformation Behavior and Damping Capacity in Fe-17Mn Alloy. Mater. Sci. Eng. A.

[114] Hornbocen E. (1985). The Effect of Variables on Martensitic Transformation Temperatures. Acta Metall..

[115] Ishida K. (1977). Effect of Alloying Elements on the Critical Driving Force of Martensitic Transformation in Iron Alloys. Scr. Metall..

[116] Kozłowska A. (2025). Dilatometric Study on Phase Transformations in Non-Deformed and Plastically Deformed Medium-Mn Multiphase Steels with Increased Al and Si Additions. J. Therm. Anal. Calorim..

[117] Yang K., Li Y., Hong Z., Du S., Ma T., Liu S., Jin X. (2021). The Dominating Role of Austenite Stability and Martensite Transformation Mechanism on the Toughness and Ductile-to-Brittle-Transition Temperature of a Quenched and Partitioned Steel. Mater. Sci. Eng. A.

[118] Zhou Q., Qian L., Tan J., Meng J., Zhang F. (2013). Inconsistent Effects of Mechanical Stability of Retained Austenite on Ductility and Toughness of Transformation-Induced Plasticity Steels. Mater. Sci. Eng. A.

[119] Wang M., Liang X., Ren W., Tong S., Sun X. (2023). Effect of Mn Content on the Toughness and Plasticity of Hot-Rolled High-Carbon Medium Manganese Steel. Materials.

[120] Wang C., Wang M., Shi J., Hui W., Dong H. (2008). Effect of Microstructural Refinement on the Toughness of Low Carbon Martensitic Steel. Scr. Mater..

[121] Calcagnotto M., Adachi Y., Ponge D., Raabe D. (2011). Deformation and Fracture Mechanisms in Fine- and Ultrafine-Grained Ferrite/Martensite Dual-Phase Steels and the Effect of Aging. Acta Mater..

[122] Du H., Gong Y., Liang T., Yuantao X., Lu X., Zeng Q., Jin X. (2020). Enhancement of Impact Toughness Via Tailoring Deformation Compatibility of Constituent Phases in Duplex Q&P Steel with Excellent Strength and Ductility. Metall. Mater. Trans. A.

[123] Capdevila C., Caballero F.G., García De Andrés C. (2003). Analysis of Effect of Alloying Elements on Martensite Start Temperature of Steels. Mater. Sci. Technol..

[124] Hou Y., Hou Z., Liu Y., Guo N., Xia Y., Yang Y., Korkolis Y.P., Wang Z., Wang J., Min J. (2025). Enhanced Strength, Hardening and Ductility Under Low Temperature Condition of an Ultra-High Strength Quenching and Partitioning Steel. J. Mater. Res. Technol..

[125] Toji Y., Nakagaito T., Matsuda H., Hasegawa K., Kaneko S. (2023). Effect of Microstructure on Mechanical Properties of Quenching and Partitioning Steel. ISIJ Int..

[126] Deng B., Yang D., Wang G., Hou Z., Yi H. (2021). Effects of austenitizing temperature on tensile and impact properties of a martensitic stainless steel containing metastable retained austenite. Materials.

[127] Wojtacha A., Kozłowska A., Skowronek A., Gulbay O., Opara J., Gramlich A., Matus K. (2025). Design Concept and Phase Transformation Study of Advanced Bainitic-Austenitic Medium-Mn Steel. Sci. Rep..

[128] Kozłowska A., Janik A., Radwanski K., Grajcar A. (2019). Microstructure Evolution and Mechanical Stability of Retained Austenite in Medium-Mn Steel Deformed at Different Temperatures. Materials.

[129] Li K., Qian, Wei C., Yu W., Ding Y., Ren L., Chen Z., Zhang F., Meng J. (2024). Study on the Relationship Between the Microstructure Characteristics, Tensile Properties and Impact Toughness of Carbide-Free Bainitic Steel. Mater. Charact..

[130] Suh M.S., Nahm S.H., Suh C.M., Park N.K. (2022). Impact Toughness of Spring Steel after Bainite and Martensite Transformation. Metals.

[131] Zhu R., Long X., Zhang F., Yang Z., Li Y., Yang Y., Wang Y. (2025). Optimization of Alloying Element Types and Contents in High Strength and High Toughness Bainitic Steels. J. Mater. Res. Technol..

